# Hierarchical Model Selection and Control for Latency-Energy Optimization in MEC-Assisted Vehicular Networks

**DOI:** 10.3390/s26154969

**Published:** 2026-08-05

**Authors:** Inseok Song, Seungwoo Kang, Seyha Ros, Seokhoon Kim

**Affiliations:** 1 Department of Software Convergence, Soonchunhyang University, Asan 31538, Republic of Korea; sis5041@sch.ac.kr (I.S.); oooksw12@sch.ac.kr (S.K.); rosseyha003@gmail.com (S.R.); 2 Department of Computer Software Engineering, Soonchunhyang University, Asan 31538, Republic of Korea

**Keywords:** vehicular networks, multi-access edge computing, hierarchical deep reinforcement learning, AI model selection, deadline-aware latency–energy optimization

## Abstract

Multi-access edge computing (MEC) enables computation-intensive perception and decision-making tasks in vehicular networks to be offloaded to nearby edge servers. Existing approaches usually fix the artificial intelligence (AI) inference model, overlooking how model selection jointly affects latency, energy consumption, and service reliability. We propose a hierarchical model selection and control (HMSC) framework based on deep reinforcement learning (DRL) for MEC-assisted vehicular networks. The framework couples a vehicle-layer MAPPO component that provides a communication interface representation for subchannel assignment and energy accounting with a centralized MEC-layer soft actor-critic (SAC) agent that, under SDN orchestration, adaptively selects lightweight or high-fidelity AI models and allocates computational resources. Accordingly, the core contribution of this paper lies in MEC-side model-aware computation control under an explicitly defined subchannel-contention abstraction, rather than in physical-layer transmit-power optimization. Both layers are guided by a composite objective that integrates normalized end-to-end (E2E) latency, normalized energy consumption, and a deadline-violation penalty. Using a discrete-time simulation framework, HMSC reduces E2E latency compared with static inference and non-hierarchical DRL baselines and sustains a higher deadline satisfaction ratio (DSR) under constrained uplink throughput and varying traffic loads. The learned policy is load-aware, favoring high-fidelity inference under light load and lightweight inference under congestion; a post hoc analysis using YOLOv5-family accuracy reference further quantifies the inference-quality implications of this adaptive selection behavior. These results show that coordinated MEC-side control of AI model selection and computation, under a shared deadline-aware objective, provides a robust latency–energy trade-off for MEC-assisted vehicular networks.

## 1. Introduction

The proliferation of intelligent transportation systems (ITS) and autonomous vehicles has accelerated the development of vehicular networks, in which vehicles communicate with other vehicles and surrounding infrastructure through Vehicle-to-Everything (V2X) technologies [1]. To ensure safe and efficient autonomous driving, vehicular communication systems must provide ultra-reliable and low-latency connectivity as well as energy-efficient computation [2,3]. However, the inherently dynamic topology of vehicular networks, rapidly varying channel conditions, and the increasing computational demands of perception and decision-making tasks pose significant challenges to meeting these requirements [4,5].

To overcome these challenges, multi-access edge computing (MEC) has emerged as a promising paradigm for executing computation-intensive tasks at the network edge, close to vehicles. By offloading computationally intensive tasks to nearby MEC servers, vehicles can reduce end-to-end (E2E) latency while alleviating the computational burden on onboard computing units. Recent studies have investigated MEC-based task offloading, bandwidth scheduling, and joint optimization of communication and computation in vehicular networks [6,7,8]. However, most prior studies have focused mainly on resource allocation and task offloading decisions, whereas the influence of AI model characteristics on overall system performance remains insufficiently investigated. In particular, whether a lightweight or high-fidelity AI model is selected can significantly affect latency, energy efficiency, and inference accuracy, especially under congested and heterogeneous vehicular network conditions.

In modern vehicular systems, AI models play an important role in perception, cooperative sensing, and decision making. Lightweight AI models, such as efficient transformer architectures and compact neural networks, are well suited to latency-sensitive tasks because of their reduced computational complexity and lower power consumption [9]. However, their limited representational capacity can degrade perception accuracy in complex traffic scenes. In contrast, high-fidelity AI models can achieve higher inference accuracy but incur increased inference delay and energy consumption because of their greater computational demands, which is consistent with the performance characteristics observed in AI accelerator architectures [10,11]. Therefore, a trade-off exists between computational efficiency and model accuracy, and static AI model assignment may lead to suboptimal performance in dynamically changing vehicular environments.

To address this limitation, this paper proposes a hierarchical model selection and control (HMSC) framework based on deep reinforcement learning (DRL) for vehicular networks supported by MEC. The proposed framework enables adaptive selection between lightweight and high-fidelity AI models based on real-time network states, computation capacity, and energy consumption. By incorporating AI model selection into the DRL policy optimization process, the system reduces E2E delay, energy consumption, and the deadline violation rate while maintaining robust performance under network congestion and varying traffic-load conditions. The overall architecture coordinates the vehicle, MEC, and control layers: the vehicle layer provides a communication interface representation for subchannel load balancing and energy accounting, while the MEC layer performs the model-aware computation decisions that constitute the dominant source of performance gains in the present evaluation. This design intentionally separates the communication interface abstraction from the main optimization contribution: the present paper does not aim to provide a full physical-layer communication control solution, but instead studies how MEC-side AI model selection and computation resource control affect latency, energy consumption, and deadline satisfaction under a controlled and reproducible uplink-contention model. While prior MEC offloading and hierarchical DRL have addressed task scheduling, communication resource allocation, and computation offloading in vehicular environments, the role of AI model characteristics has typically been treated as either a static cost parameter or has been absent from the decision space altogether. Furthermore, existing SDN- and NFV-driven control-plane frameworks operate primarily at the flow, slice, or generalized task level, without exposing AI model selection as a network-controllable decision variable. The proposed HMSC framework differs from these prior approaches in three key technical aspects: (i) AI model selection is incorporated as a first-class MEC-side decision variable and optimized together with DVFS-based computation control within a unified deadline-aware DRL formulation; (ii) the hierarchical architecture separates the vehicle-side communication interface representation from the MEC-side SAC-based model-aware computation controller under SDN orchestration, reducing policy coupling complexity while preserving coordinated training through a shared composite objective; and (iii) the objective explicitly internalizes deadline satisfaction as a penalty term rather than treating it solely as a feasibility constraint, enabling penalty-based policy optimization in stochastic vehicular environments. These technical distinctions are detailed in the related-work analysis of Section 2 and are reflected in the three contributions summarized below. Motivated by these limitations, this study develops the HMSC framework and makes the following technical contributions:

- First, we investigate a model-aware MEC computation control problem for vehicular perception and decision-making tasks under stringent latency and energy constraints. The communication side is represented through an explicitly stated subchannel contention interface for subchannel-load balancing and energy accounting, while the main optimization target is MEC-side AI model selection and DVFS-based computation control. Accordingly, the measured performance improvements are primarily attributed to the MEC-side model-aware SAC controller rather than to physical-layer transmit-power optimization.
- Second, we integrate AI model selection into the DRL policy learning process, enabling the proposed framework to select an appropriate AI model according to real-time MEC workload, queue state, computational capacity, and energy consumption. We formulate a deadline-aware model selection and computation control problem that minimizes a composite objective comprising E2E delay, total energy consumption, and deadline violation, thereby accounting for both the efficiency and reliability requirements of safety-critical vehicular applications. In the present evaluation, the demonstrated gains are attributed primarily to the MEC-side SAC agent for AI model selection and DVFS control. The vehicle-side MAPPO component is retained as a common communication interface representation for subchannel assignment and energy accounting, and is not claimed as a source of transmission-power-induced rate optimization gain.
- Finally, we develop a discrete-time simulation framework that captures V2X communication, task arrivals, MEC-based AI inference, and hierarchical DRL-based control processes. Extensive simulation experiments are conducted to evaluate the proposed framework, showing that the proposed approach achieves consistent improvements over baseline methods in terms of E2E latency, energy efficiency, and service reliability under varying numbers of vehicles, task data sizes, and channel conditions.

The rest of this paper is structured as follows. Section 2 discusses prior studies on MEC-assisted vehicular networks and DRL-based resource optimization. Section 3 defines the system model and formulates the optimization problem. Section 4 presents the proposed DRL-based algorithms for model-selection control and resource allocation. Section 5 describes the simulation environment and analyzes the performance results. Finally, Section 6 summarizes the main findings and outlines future research directions.

## 2. Related Works

In recent years, MEC has become a key enabling paradigm for vehicular edge computing (VEC) by bringing computing resources closer to vehicles, thereby alleviating onboard computational limitations and supporting stringent real-time latency requirements. As autonomous driving applications increasingly rely on real-time perception and decision-making, recent studies have investigated computation offloading, V2X communication, and resource management to support latency-sensitive service in MEC-assisted vehicular systems [12,13]. These works highlight the importance of jointly optimizing communication and computation operations in dynamic vehicular environments and form the foundation for advanced control frameworks based on DRL and software-defined networking (SDN).

### 2.1. MEC-Assisted Vehicular Networks and Task Offloading

A substantial body of research has examined task offloading and cooperative scheduling in MEC-assisted V2X architectures, where vehicles, RSUs, and MEC servers jointly process computation-intensive workloads [14,15,16]. Nash-equilibrium-based schemes show that the joint optimization of task assignment and routing can significantly reduce delay and energy consumption [14]. Beyond single-layer approaches, multi-layer optimization frameworks adopt alternating direction method of multipliers (ADMM)-based mechanisms and hierarchical aerial MEC structures to coordinate computation and communication resource across heterogeneous network layers [16,17]. Recent studies further incorporate task prioritization, integrated sensing and communication (ISAC)-assisted sensing, ultra-reliable low-latency communication (URLLC)-aware resource allocation, and velocity-adaptive access control to address diverse quality of service (QoS), fairness, and information-freshness requirements in safety-critical vehicular environments [18,19,20]. Xu et al. investigated a velocity-adaptive access scheme for semantic-aware vehicular networks, jointly optimizing access fairness and age of information (AoI) in 5G NR V2X Mode 2 [21]. However, most of these works assume a fixed AI inference pipeline or treat model complexity as a static cost parameter, without incorporating AI model selection as part of the joint MEC-side computation-control problem.

### 2.2. DRL and SDN-Enabled Resource Management for VEC

DRL has emerged as a promising approach for dynamic task scheduling and resource allocation in VEC [22,23]. DRL-based approaches jointly optimize computation offloading decisions, communication resource allocation, and computing resource scheduling to reduce delay and energy consumption in highly dynamic internet of vehicles (IoV) and VEC environments [24,25,26]. Multi-agent reinforcement learning further enables cooperation among distributed vehicles and edge servers, supporting stability-aware and coordinated offloading strategies and ensuring queue stability in B5G/6G networks [27,28]. At the network control plane, SDN provides logically centralized and programmable control, while network function virtualization (NFV) enables flexible deployment of virtualized network functions across edge nodes; together, they support data-driven resource orchestration in MEC-assisted systems [29,30]. Representative SDN- and NFV-enabled studies include graph-based open RAN (O-RAN) control for real-time AI workload management and DRL-driven service function chaining (SFC) for dynamic MEC service placement in IoT networks [31,32]. Additional works investigate QoS-driven slicing management for vehicular communications, large-scale SFC orchestration in smart-city core networks, and DRL-based backbone SDN control in UAV-assisted networks for computational resource efficiency [33,34,35]. These studies collectively demonstrate the effectiveness of centrally coordinated, learning-driven control across heterogeneous edge resources. However, they primarily operate at the flow, slice, or generalized task level and do not incorporate AI model selection at the MEC server. Moreover, existing DRL- and SDN-based frameworks lack a unified architecture that exposes AI model selection as an explicit MEC-side control variable and coordinates it with computation resource allocation under a hierarchical vehicle-edge control structure.

### 2.3. Edge Intelligence and Distributed AI Workloads

Research on distributed intelligence and federated learning has addressed the challenges of deploying and optimizing AI workloads across resource-constrained edge-cloud environments, particularly with respect to communication overhead, latency, and resource heterogeneity [36,37,38]. Additional studies have explored graph-based and DRL-based orchestration mechanisms for O-RAN resource management, as well as DRL-based AI workload scheduling and collaborative inference in MEC/edge environments [39,40,41]. However, although these studies address collaborative edge intelligence, vehicular DNN inference, and latency-oriented scheduling, they do not explicitly investigate how adaptive AI model selection affects the joint latency–energy trade-off in MEC-assisted vehicular perception and decision-making pipelines. Existing studies have not fully addressed the joint integration of (i) a vehicle-side communication interface representation for subchannel assignment and energy accounting, (ii) AI model selection between lightweight and high-fidelity models at the MEC layer, and (iii) SDN-enabled centralized orchestration within a unified control structure. To address these gaps, the proposed framework adopts a three-layer architecture in which MAPPO agents at the vehicle layer provide a communication interface representation, an SAC agent at the MEC layer performs model-aware computation control, and an SDN controller coordinates state aggregation and policy enforcement to improve latency, energy efficiency, and robustness in MEC-assisted vehicular networks.

## 3. System Models

**Figure 1:**
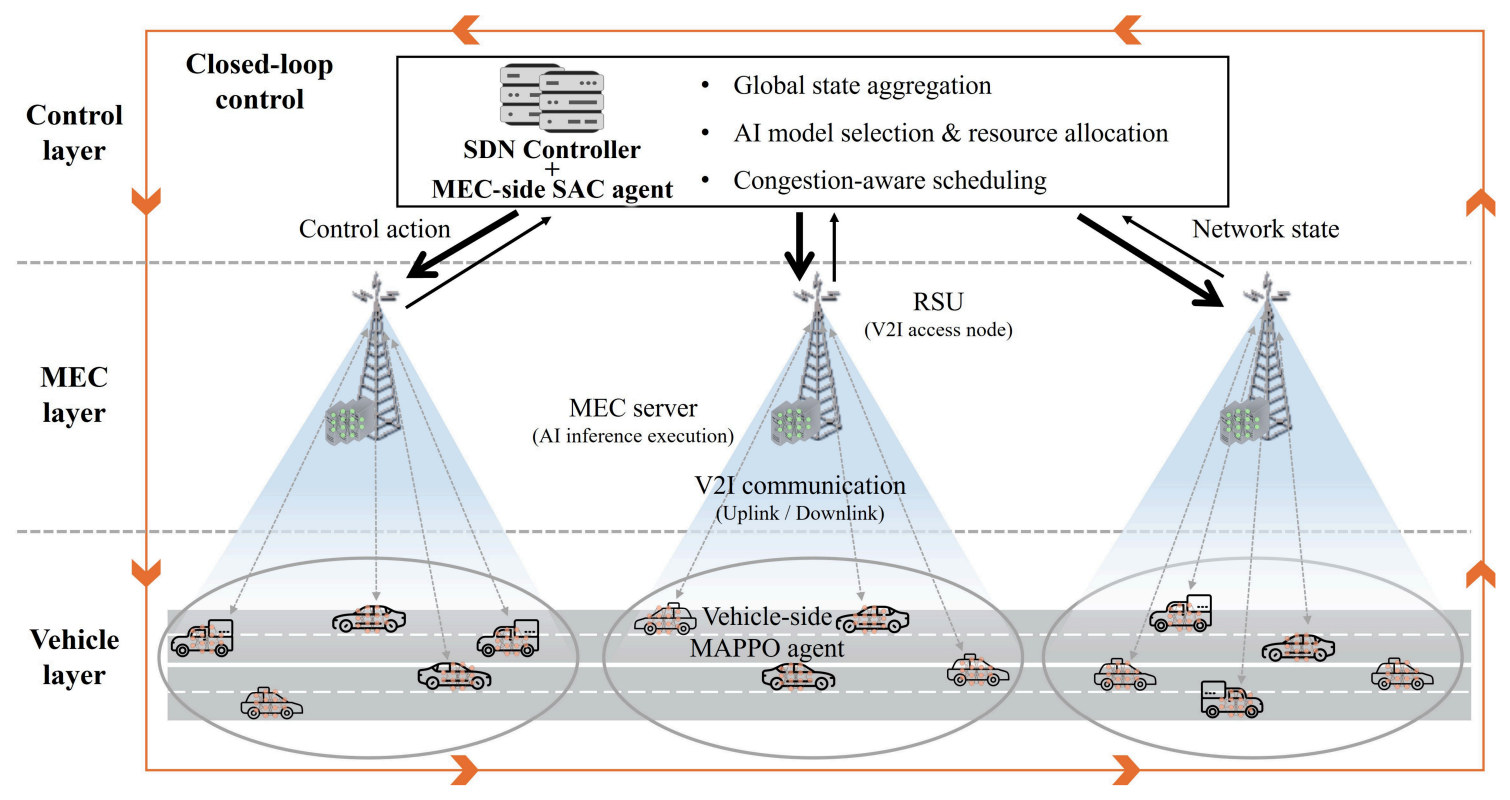
System architecture of the proposed HMSC framework in MEC-assisted vehicular networks.

In this section, we describe the MEC-assisted vehicular network architecture considered in this work. As illustrated in Figure 1, vehicles generate perception tasks and offload the corresponding task data to the nearest RSU via V2X links, while each RSU forwards the received data to its associated MEC server for processing. MEC servers execute AI inference using either lightweight or high-fidelity AI models, where the model type is adaptively selected by a DRL-based controller according to the current load and computation state. A centralized SDN controller aggregates network states from all RSUs and coordinates traffic forwarding, state aggregation, and policy enforcement to maintain stable system operation. Table 1 lists the main notation and parameters adopted in the system formulation.

### 3.1. Network Architecture

As shown in Figure 1, the considered vehicular network consists of vehicles, RSUs, MEC servers, and a centralized SDN controller. More specifically, let V denote the set of vehicles served within the RSU coverage area. Each vehicle periodically generates perception and decision-making tasks and offloads the corresponding task data to the nearest RSU through a V2X uplink. The RSUs serve as wireless access points and relay nodes, forwarding the received tasks to their associated MEC servers via high-capacity backhaul connections. Each MEC server performs task processing and AI inference using either a lightweight or high-fidelity model. Within the proposed HMSC framework, the model type to be executed on each MEC server is adaptively selected by a centralized MEC-side SAC agent logically located in the control layer, operating under SDN orchestration. As shown in Figure 1, the centralized SDN controller and the MEC-side SAC agent are jointly located in the control layer, while the MEC servers performing AI inference reside in the MEC layer. The agent observes aggregated workload, queue states, resource utilization, and task arrival information collected from the RSUs and MEC servers. The SDN controller aggregates this network state information and supports congestion-aware coordination, state aggregation, AI model selection, and computational resource allocation across the network. Under this hierarchical structure, control decisions are represented at two layers. At the vehicle layer, multi-agent MAPPO agents provide interface variables for subchannel assignment and energy accounting, while at the MEC layer, the centralized SAC agent performs AI model selection and computational resource allocation. This architecture enables coordinated vehicle-edge operation, with the present evaluation focusing on MEC-side AI model selection and computation control under a common uplink configuration.

### 3.2. Communication Model

We consider a wireless uplink from each vehicle to its serving RSU, followed by a backhaul connection to the co-located MEC server. Vehicles share K orthogonal OFDM subchannels; let $n_{c}(t)$ denote the number of vehicles on subchannel c at slot t under the MAPPO channel-selection policy. Rather than resolving per-vehicle SINR explicitly, we adopt a phenomenological multi-user contention model in which the realized uplink rate is

(1) $$R_{v,r}\left(t\right)=B_{UL}\;\cdot \;{\left[1+\log_{2}n_{c}(t)\right]}^{-1}\;\cdot \;\eta ,$$

where $B_{UL}$ is the per-subchannel aggregate uplink capacity, η∈{1.0,0.5} is an effective channel-quality factor (normal/degraded), and the $\log_{2}n_{c}(t)$ term captures sublinear throughput degradation as more vehicles contend for one subchannel. This model is deliberately conservative: adding a second vehicle to an already occupied subchannel halves the per-vehicle rate, a pessimistic bound relative to Shannon capacity. It isolates the two contention effects central to this study—channel selection load balancing across subchannels and per-subchannel multi-user sharing—without introducing SINR parameters orthogonal to the hierarchical control contribution and difficult to reproduce across snapshot densities. All vehicles assigned to subchannel c in slot t obtain the identical realized rate in (1); no per-vehicle SINR is computed and no additional intra-subchannel time- or resource-block scheduling is applied, so multi-user contention is captured exclusively and only once by the ${\left[1+{log}_{2}n_{c}(t)\right]}^{-1}$ sharing factor. For task i generated by vehicle $v_{i}$, the selected subchannel is $c_{i}\left(t\right)=c_{v_{i}}(t)$, and $n_{c_{i}}(t)$ denotes the number of vehicles assigned to the same subchannel at slot t. The uplink delay then follows directly from (1) as

(2) $$T_{i}^{ul}=\frac{D_{i}}{R_{v_{i},r}(t)}=\frac{D_{i}\;\cdot \;[1+\log_{2}n_{c_{i}}(t)]}{B_{UL}\;\cdot \;\eta }.$$

Because $p_{v}(t)$ does not appear in (1) or (2), the present evaluation does not claim that transmit power control improves the uplink rate or E2E latency. The power variable is retained for transmission energy accounting in (11) and for compatibility with future power- and SINR-dependent extensions. Therefore, the communication model is used as a controlled and reproducible contention abstraction for evaluating MEC-side model-selection and computation-control decisions, rather than as a realistic physical-layer model for optimizing transmit power, SINR, or link adaptation.

After receiving the task, RSU r forwards the data to its co-located MEC server through a high-capacity backhaul link. Since the backhaul link provides abundant capacity relative to the wireless access link, the backhaul forwarding delay is modeled as a constant value $\tau_{bh}$, as expressed in (3),

(3) $$T_{i}^{bh}=\tau_{bh},$$

where $\tau_{bh}$ denotes the fixed backhaul delay between the RSU and its co-located MEC server.

Finally, the computation result, whose output size $D_{i}^{res}$ is much smaller than the input size $D_{i}$, is sent back from the MEC server to the vehicle through the RSU. Since the output data size is small, the downlink delay $T_{i}^{dl}$ is modeled as a constant value. Therefore, the total communication delay of task i is expressed as

(4) $$T_{i}^{comm}=T_{i}^{ul}+T_{i}^{bh}+T_{i}^{dl}.$$

### 3.3. Computation Model

Each task i generated by vehicle v is characterized by an input data size $D_{i}$ and a model-dependent computation requirement $C_{i}^{\left(m\right)}$, where m∈{L, H} denotes the selected AI model type. Here, L and H represent the lightweight and high-fidelity models, respectively. The lightweight model requires fewer CPU cycles because of its compact structure, whereas the high-fidelity model provides higher inference accuracy at the cost of increased computational complexity.

Let $f_{e}^{max}$ denote the maximum CPU frequency of MEC server e, and let $f_{e,i}$ denote the CPU frequency allocated to task i. The computation delay of task i processed with model m is given by

(5) $$T_{i}^{comp}(m)=\frac{C_{i}^{\left(m\right)}}{f_{e,i}}.$$

Based on a standard dynamic voltage and frequency scaling (DVFS) model, the computation energy consumed by MEC server e to execute task i is expressed as

(6) $$E_{i}^{comp}\left(m\right)=\kappa {\left(f_{e,i}\right)}^{2}C_{i}^{\left(m\right)},$$

where κ is an effective switched capacitance coefficient of the MEC processor. The two AI model classes considered in this work are intended to represent practical lightweight and high-fidelity object detection models commonly deployed in vehicular perception pipelines. As reference values, the lightweight class corresponds to compact detectors such as YOLOv5n (4.5 GFLOPs, 1.9 M parameters, ${mAP}_{50}=0.457$), while the high-fidelity class corresponds to larger detectors such as YOLOv5l (109.1 GFLOPs, 46.5 M parameters, ${mAP}_{50}=0.673$) on the COCO val2017 benchmark at 640×640 input resolution [42]. This corresponds to an inference-accuracy gap of approximately 21.6 percentage points between the two classes, accompanied by a roughly 24-fold difference in computational cost. The cycle-based parameters in Table 2 ($C_{L}=30\times 10^{6}$ and $C_{H}=100\times 10^{6}$ cycles per task) are chosen to preserve the qualitative lightweight/high-fidelity ordering of this model pair while keeping the simulated per-task computation load within the capacity regime of the modeled MEC server; the exact 24-fold GFLOPs ratio is not reproduced at the cycle level, as doing so would render high-fidelity inference infeasible within the 1.0 s deadline under the evaluated load range. Equivalently, $T_{i}^{comp}(m)={cmp}_{m}/f_{GHz}$ with ${cmp}_{L}=30\;\mathrm{ms}$, ${cmp}_{H}=100\;\mathrm{ms}$ at 1 GHz, matching $C_{L}=30\times 10^{6}$, $C_{H}=100\times 10^{6}$ cycles. Accordingly, the choice of m∈{L, H} reflects a network-controllable trade-off between computation cost (latency, energy) and inference quality, with the following inequalities holding consistently:

(7) $$C_{i}^{\left(H\right)}>C_{i}^{\left(L\right)},\;{\;T}_{i}^{comp}(H)>T_{i}^{comp}(L),\;{\;E}_{i}^{comp}(H)>E_{i}^{comp}(L),$$

which captures the higher computation load, energy demand, and inference quality of the high-fidelity model.

MEC queue dynamics and resource allocation. At each time slot ∆t, newly arrived tasks generated under the Bernoulli arrival process described in Section 5.1 join the per-MEC task queue and are served in first-in-first-out (FIFO) order. Let $A_{e}(t)$, $C_{e}(t)$, and $D_{e}(t)$ denote the sets of tasks newly arriving at MEC server e, completing service, and expiring or being dropped during slot t, respectively. The queue-set evolution can be written as

(8) $$Q_{e}(t+1)={\left[Q_{e}(t)\cup A_{e}(t)-C_{e}(t)-D_{e}(t)\right]}_{FIFO}.$$

Equivalently, the scalar queue length used in the MEC state is obtained as $Q_{e}(t+1)=\left|Q_{e}(t+1)\right|=\left|\left\{i\in {pending}_{e}(t+1)\;:\;{deadline}_{i}>t+1\right\}\right|$.

Up to $N_{core}$ tasks execute concurrently, and each task holds its DVFS-allocated frequency $f_{e,i}$ for the duration of its computation $T_{i}^{comp}(m)$, after which the core is released for subsequent tasks. Concurrency is bounded by $N_{core}=4$ via an earliest-available multi-core scheduler. Constraint C2b constrains the aggregate DVFS allocation rather than the concurrent task count; in this setting, $\left(F_{e}^{max}=20\;\mathrm{GHz}=N_{core}\times f^{max}\right)$. The SDN controller polls aggregate state $\left\{Q_{e}(t),\;U_{e}^{CPU}(t),\;\lambda_{e}(t),\;T_{e}^{queue}(t)\right\}$ at the beginning of each time slot ∆t=1.0 s; the resulting inter-layer control signaling delay is in the order of milliseconds in practical SDN deployments and is therefore negligible compared to the per-task deadline $T_{max}=1.0\;s$. This assumption is consistent with standard SDN architectures in which controller processing delays are typically in the order of 0.1−1 ms in intra-data-center settings.

MEC servers maintain task queues, and the queueing delay of task i is denoted by $T_{i}^{queue}$. The total MEC processing delay of task i is expressed as

(9) $$T_{i}^{MEC}=T_{i}^{queue}+T_{i}^{comp}(m).$$

A DRL agent operating at the MEC layer adaptively selects the AI model type m and allocates computational resource based on the observed network and system state, including queue lengths, CPU utilization, task arrival rates, and subchannel-occupancy indicators. By jointly considering latency, energy consumption, and workload conditions, the agent determines model selection and resource allocation strategies to improve inference efficiency and overall system performance.

### 3.4. Latency and Energy Model

For each task i generated by vehicle v, the E2E delay consists of the uplink transmission delay, backhaul transmission delay, downlink delay, MEC queueing delay, and computation delay. Based on the communication and computation models described above, the E2E delay of task i is expressed as

(10) $$T_{i}^{E2E}=T_{i}^{ul}+T_{i}^{bh}+T_{i}^{MEC}+T_{i}^{dl},$$

where $T_{i}^{MEC}$ includes the MEC queueing delay and computation delay, and $T_{i}^{dl}$ denotes the downlink delay for returning the computation result to the vehicle.

The total energy consumption of task i includes the uplink transmission energy at the vehicle and the computation energy at the MEC server. Assuming that the selected transmit power remains constant during the uplink transmission interval of each task, the uplink transmission energy is given by

(11) $$E_{i}^{tx}=p_{v}(t)T_{i}^{ul},$$

where $p_{v}(t)$ denotes the transmit power of vehicle v selected at time slot t. Since the computation energy $E_{i}^{comp}(m)$ has already been defined by the DVFS-based model in Equation (6), the total energy consumption of task i is expressed as

(12) $$E_{i}^{tot}=E_{i}^{tx}+E_{i}^{comp}(m).$$

For latency and energy comparisons, we report completed-task averages because E2E latency and realized computation energy are fully observed only for tasks that are completed within the simulation horizon. Let C denote the set of tasks completed within the simulation horizon. The completed-task average E2E delay is computed as

(13) $${\bar{T}}_{comp}=\frac{1}{\left|C\right|}\sum_{i\in C}T_{i}^{E2E},$$

and the completed-task average energy consumption is calculated as

(14) $${\bar{E}}_{comp}=\frac{1}{\left|C\right|}\sum_{i\in C}E_{i}^{tot}.$$

To avoid bias caused by completed-task-only averaging, we additionally report arrival-based energy, completion rate, drop rate, and throughput in Section 5.5.1.

We also consider the deadline satisfaction ratio (DSR), which measures the fraction of generated tasks that are completed within the common latency deadline and is defined as

(15) $$DSR=\frac{1}{N}\sum_{i=1}^{N}1\{{i\in C,T}_{i}^{E2E}\leq T_{max}\},$$

where N denotes the total number of generated tasks, $T_{max}$ denotes the common latency deadline, and 1{·} is the indicator function.

### 3.5. Problem Formulation

Based on the system models described above, we formulate a composite optimization problem that jointly addresses latency, energy, and deadline satisfaction in MEC-assisted vehicular networks. Let I denote the set of perception tasks generated within a decision epoch. For each task i∈I, the E2E delay $T_{i}^{E2E}$ and the total energy consumption $E_{i}^{tot}$ are defined in Section 3.4. In the proposed HMSC framework, the vehicle layer provides communication interface variables, collectively denoted by $a_{comm}(t)={\left\{[p_{v}(t),\;c_{v}(t)]\right\}}_{v\in V}$, which determine subchannel occupancy and transmission-energy accounting in the simulator. The centralized MEC-side agent selects a slot-level AI model class $m\left(t\right)\in \{L,\;H\}$ and a slot-level DVFS frequency $f_{e}(t)$. In the present evaluation, the optimization focus is on MEC-side model selection and computation control conditioned on the common vehicle-side uplink configuration. These slot-level decisions are then dispatched by the SDN controller to the tasks arriving in the current slot according to the implementation rule $m_{i}(t)=m(t)$, $f_{e,i}\left(t\right)=f_{e}\left(t\right)$, ∀i∈I(t), where I(t) denotes the set of tasks arriving at slot t. Thus, the per-task variables $m_{i}(t)$ and $f_{e,i}\left(t\right)$ are induced by the slot-level MEC decision rather than independently optimized for each task. To capture the joint objective over latency, energy, and deadline satisfaction, we define the following composite cost function

(16) $$J=\frac{1}{\left|I\right|}\sum_{i\in I}\left(w_{T}{\tilde{T}}_{i}+w_{E}{\tilde{E}}_{i}\right)+\beta (1-DSR),$$

In (16), ${\tilde{T}}_{i}$ and ${\tilde{E}}_{i}$ denote the normalized E2E delay and total energy of task i, respectively, and are defined as ${\tilde{T}}_{i}=T_{i}^{E2E}/T_{max}$ and ${\tilde{E}}_{i}=E_{i}^{tot}/E_{ref}$, where $T_{max}$ is the task deadline and $E_{ref}$ is a reference energy bound. The reference energy $E_{ref}=0.3$ J was set based on the upper envelope of total per-task energy observed in preliminary simulation runs under the highest-load scenario, so that ${\tilde{E}}_{i}$ remains approximately within [0, 1] during training. The weighting coefficients $w_{T}$ and $w_{E}$ control the relative emphasis between latency and energy consumption, while β determines the penalty for deadline violation. In this work, we set $w_{T}=w_{E}=1.0$ to provide a balanced latency–energy trade-off and β=8.0 to strongly enforce deadline satisfaction, reflecting the safety-critical requirements of real-time vehicular applications; this value was selected through preliminary tuning to sufficiently penalize deadline violations relative to the normalized latency and energy terms. To separately examine the intrinsic latency–energy trade-off during evaluation, we additionally define a post hoc sensitivity cost using a normalized weighting factor α∈[0,1], excluding the deadline violation penalty term. In the per-step reward computation used in Algorithms 1 and 2, the system-level deadline penalty term $\beta \left(1-DSR\right)$ in (16) is implemented through a per-task deadline violation indicator, i.e., $\beta \cdot 1\{T_{i}^{E2E}>T_{max}\}$. This implementation is consistent with the system-level form in (16) when averaged over the task set I. During implementation, this cost is evaluated over the task outcomes finalized at each slot, while arrival-based reliability statistics are separately reported in Section 5.5.1 to account for incomplete and dropped tasks.
**Algorithm 1:** Vehicle-Side MAPPO-Based Communication Interface1: Input: Set of vehicles V, RSUs R, fixed V2X channel bandwidth $B_{c}$, initial actor parameters $\left\{\theta_{v}\right\}$, and centralized critic parameters φ2: Output: Trained vehicle policies $\left\{\pi_{\theta_{v}}\right\}$
3: Initialize decentralized actor networks
$\left\{\pi_{\theta_{v}}\right\}$ and centralized critic
$V_{\varphi }$
4: for each training episode do5: Initialize the environment state and clear the trajectory buffer D
6: for each time slot t do7: Each vehicle
v observes its local state $s_{v}(t)$
8: Each vehicle v outputs the interface action $a_{v}(t)=[p_{v}(t),\;c_{v}(t)]$, where $p_{v}(t)$ is usedfor energy accounting and $c_{v}(t)$ determines the subchannel index9: Apply the selected subchannel indices to determine $n_{c}(t)$, compute the uplink rate using (1), and observe the next state $s_{v}(t+1)$
10: Compute task-level delay and energy according to (10) and (12)11: Wait for the MEC-layer SAC agent (Algorithm 2) to complete its model selection and CPU allocation for the current slot’s tasks; then compute the reward $r_{t}=-J$ according to (16), incorporating the normalized latency, energy, and deadline-violation terms evaluated on the slot’s completed task outcomes12: Store the transition
$\left({\{s}_{v}\left(t\right)\right\},\;\{a_{v}\left(t\right)\},\;r_{t},\;{\{s}_{v}\left(t+1\right)\})$ in
D
13:    end for14: Estimate advantages using the centralized critic and generalized advantage estimation (GAE)15: Update critic parameters φ by minimizing the value loss16: Update actor parameters $\left\{\theta_{v}\right\}$ using the MAPPO surrogate objective17: end for18: Deploy trained vehicle policies for online execution


**Algorithm 2:** SAC-Based Model-Aware Computation and Load Balancing1: Input: MEC servers
E, model set
{L, H}, actor
θ, twin critics
$\xi_{1}$,
$\xi_{2}$, targets
$\xi_{1}\prime$,
$\xi_{2}\prime$, entropy temperature
$\alpha_{ent}$, soft-update rate
τ
2: Output: Trained computation policy $\pi_{\theta }$
3: Initialize actor
$\pi_{\theta }$, twin critics
$Q_{\xi_{1}}$,
$Q_{\xi_{2}}$, and targets
$\xi_{1}\prime$
$,\;\xi_{2}\prime$
4: for each training episode do5:    Initialize the environment6:    for each time slot t do7:        SDN controller collects aggregate state $s_{e}(t)$ from all e∈E as defined in (19)8:        Sample $a_{e}\left(t\right)=[m\left(t\right),\;f_{e}(t)]$ from
$\pi_{\theta }$
9:        Apply the single decision to all tasks arriving in slot
t
10:      Observe the next states $s_{e}(t+1)$
11:      Compute reward $r_{t}=-J$ according to (16) from the slot’s completed-task outcomes12:      Form the single transition $\left(s_{e}\left(t\right),\;a_{e}(t),\;r_{t},\;s_{e}(t+1)\right)$
13:      Update twin critics $\xi_{1}$$,\;\xi_{2}$ by minimizing the soft Bellman residual
14:      Update actor θ by maximizing the entropy-regularized objective
15:      Soft-update targets $\xi_{k}^{\prime }⟵{\tau \xi }_{k}+(1-\tau )\xi_{k}\prime$, ∀k∈{1, 2}16:    end for17: end for18: Deploy trained policy $\pi_{\theta }$ for online execution.


The optimization problem is then formulated as

$$P1:\;\underset{\left\{m(t),\;f_{e}(t)\right\}}{\min}J(a_{comm}(t),\;m(t),\;f_{e}(t))$$

subject to

- $C1:f_{e}(t)\in F$, F={1,2,3,4,5} GHz,
- $C2a:\left|S_{e}\left(t\right)\right|\leq N_{core}$,
- $C2b:\left|S_{e}\left(t\right)\right|f_{e}(t)\leq F_{e}^{max}$, ∀e∈E,
- $C3:m\left(t\right)\in \{L,H\}$,
- C4 (conditioned interface variable)$:0\leq p_{v}(t)\leq p_{v}^{max},\forall v\in V$,
- $C5:T_{i}^{E2E}\leq T_{max},\forall i\in I$.

Here, $S_{e}\left(t\right)$ denotes the set of tasks concurrently admitted to service at MEC server e during slot t. Constraint C2a limits the number of simultaneously processed tasks by the number of MEC cores, while C2b ensures that the aggregate DVFS allocation of the concurrent service set does not exceed the MEC capacity. Since the implementation applies a single DVFS level $f_{e}(t)$ to all concurrently served tasks, C2b reduces to $\left|S_{e}\left(t\right)\right|f_{e}(t)\leq F_{e}^{max}$. The task-level quantities $m_{i}(t)$ and $f_{e,i}(t)$ appearing in the delay and energy expressions are obtained through the dispatch rule $m_{i}(t)=m\left(t\right)$ and $f_{e,i}(t)=f_{e}(t)$.

The composite objective J in (16) jointly accounts for E2E delay, total energy consumption, and deadline violation, thereby integrating efficiency and reliability requirements within a single optimization formulation. Constraint C5 is formulated as a per-task hard latency requirement at the optimization-problem level. However, in stochastic vehicular environments characterized by time-varying channels, random task arrivals, and queueing dynamics, exact per-task feasibility cannot be guaranteed in advance of action execution; projection-based constrained optimization is therefore intractable for real-time control. To resolve this gap between the optimization formulation and the learning procedure, we interpret C5 in a chance-constrained manner during DRL training. $Pr[T_{i}^{E2E}\leq T_{max}]\geq 1-\epsilon ,\;\forall i\in I$, where $T_{i}^{E2E}$ is the end-to-end delay of task i defined in (10), $T_{max}$ is the common latency deadline, I is the set of perception tasks generated within a decision epoch, and ϵ∈[0,1] is a small tolerance representing the maximum acceptable deadline violation probability. The penalty term β(1−DSR) in the composite objective (16) serves as an empirical penalty-based approximation of this chance-constrained deadline requirement. This construction is not an exact Lagrangian relaxation of the per-task chance constraint, and β=8.0 does not certify a specific tolerance ϵ; β was selected empirically so that the violation penalty dominated the normalized latency–energy terms in high-violation regimes, thereby encouraging but not certifying a low deadline-violation probability under this chance-constrained interpretation. This penalty-based approximation preserves gradient-based policy learning while approximating the hard constraint C5 in expectation over the task population, consistent with the safety-critical reliability requirements of real-time vehicular applications. All delay and energy terms appearing in the objective function and constraints are defined in Section 3.2, Section 3.3 and Section 3.4. Constraint C1 restricts the feasible slot-level DVFS frequency selected by the MEC-side agent, while C2a and C2b jointly enforce the multi-core service limit and the aggregate MEC CPU-capacity limit. C3 enforces the slot-level discrete AI model selection, and C4 limits the transmission power of each vehicle. Constraint C4 specifies the feasible operating range of the vehicle-side power variable used for transmission-energy accounting; it is not used to claim transmission-power-induced rate optimization in the present evaluation. C5 imposes the latency deadline requirement for each task after the slot-level model and DVFS decisions are dispatched to individual tasks.

The optimization problem P1 is a stochastic mixed discrete nonlinear decision program because it involves discrete AI model selection, DVFS-frequency selection, conditioned vehicle-side interface variables, and nonlinear coupling among delay, energy, queueing, and deadline satisfaction terms. Moreover, vehicular traffic loads, effective channel conditions, queue variations, and stochastic task arrivals make the optimal decision highly dependent on the current network and system state. Therefore, obtaining the optimal solution through conventional optimization methods is impractical for real-time vehicular network control. In the next section, we develop a hierarchical DRL framework that combines a vehicle-side communication interface representation with MEC-side AI model selection and computational resource allocation policies to minimize the cost J.

## 4. Methodology

In this section, we present the proposed hierarchical DRL-based HMSC algorithm for solving the composite optimization problem formulated in Section 3.5. The optimization problem involves a vehicle-side communication interface representation, AI model selection, and computational resource allocation under dynamic vehicular traffic loads, stochastic wireless channels, queue variations, and time-varying task workloads. Since obtaining the optimal solution through conventional optimization methods is impractical for real-time vehicular network control, the proposed HMSC framework decomposes the joint decision-making process into coordinated learning procedures at the vehicle and MEC layers.

**Figure 2:**
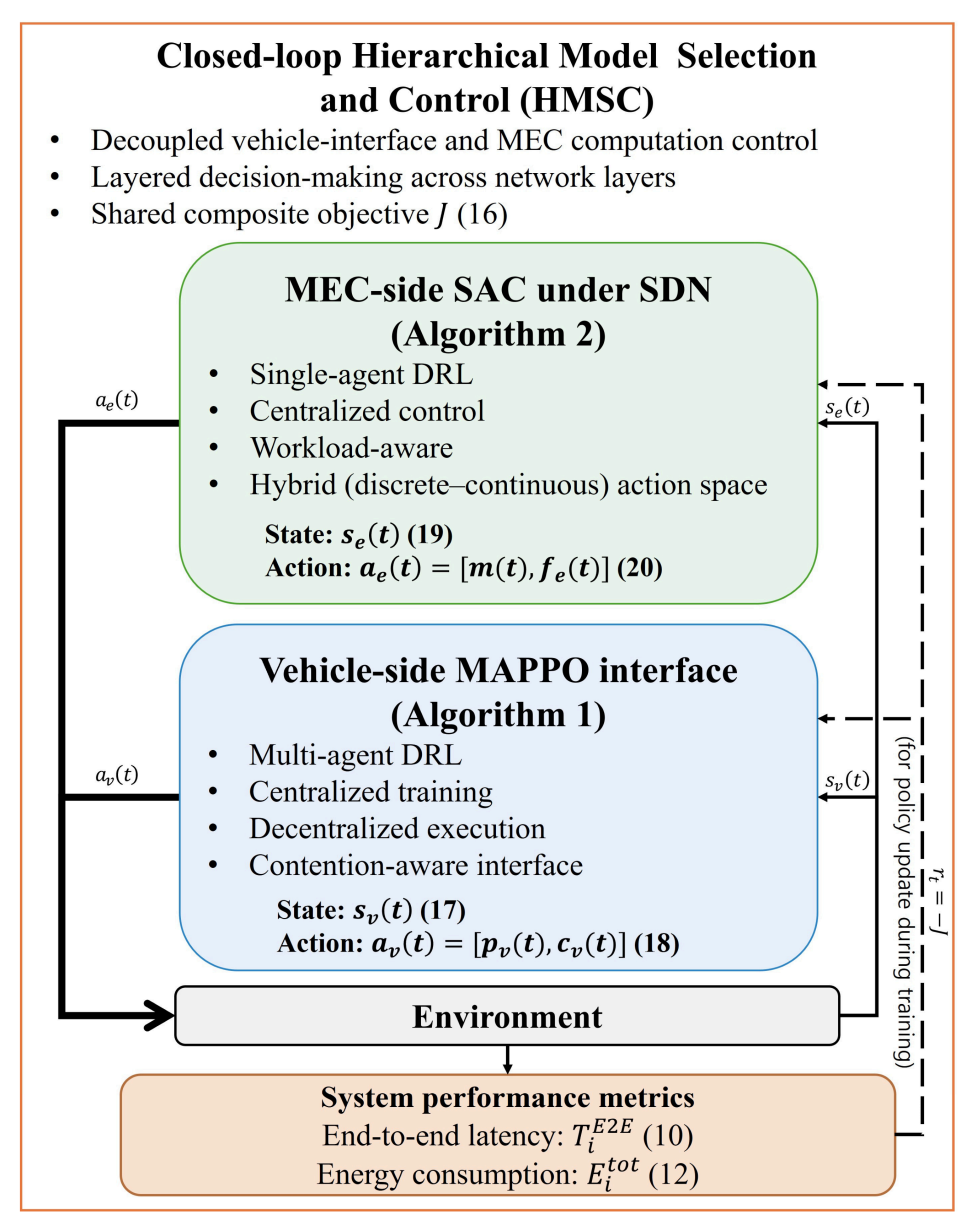
Hierarchical deep reinforcement learning framework for vehicle-edge coordination and MEC-side model-aware computation control in MEC-assisted vehicular networks.

As illustrated in Figure 2, the hierarchical DRL structure consists of vehicle-side MAPPO agents and a centralized MEC-side SAC agent. MAPPO is used for the vehicle-side multi-agent interface because it supports centralized training with decentralized execution among vehicles. SAC is used for the MEC-side controller because the MEC action contains both a discrete model-selection component and a DVFS-frequency component. In contrast, PPO is used only for the Flat-DRL baseline described in Section 5.2. Thus, MAPPO, SAC, and PPO have distinct roles in this paper: vehicle-side communication interface representation, MEC-side model-aware computation control, and non-hierarchical MEC-side baseline learning, respectively. The reward feedback is used for policy optimization during training and does not constitute a real-time operational control signal within the closed-loop architecture shown in Figure 1. This hierarchical design improves scalability, learning stability, and overall system efficiency in MEC-assisted vehicular networks.

### 4.1. Vehicle-Side Learning for Communication Interface

At the vehicle layer, we retain a MAPPO-based communication interface representation to model decentralized vehicle-side interaction with shared V2X uplink resource. In the current phenomenological rate model, vehicle-side actions provide subchannel-assignment and energy-accounting variables rather than transmission-power-induced rate optimization. This design keeps the hierarchical vehicle-edge structure explicit while ensuring that the performance gains reported in this paper are attributed to the MEC-side model-aware computation controller.

#### 4.1.1. State Representation of Vehicle

The state observed by vehicle v at time slot t is defined as

(17) $$s_{v}(t)=[h_{v,r}(t),\;I_{r}(t),\;Q_{r}(t),\;R_{v,r}(t)],$$

where $h_{v,r}(t)$ denotes the observed uplink channel indicator between vehicle v and its serving RSU r, $I_{r}(t)$ represents an aggregate co-channel load indicator at RSU r, $Q_{r}(t)$ denotes the queue length information of RSU r provided to the vehicle as a local congestion indicator, and $R_{v,r}(t)$ denotes the realized uplink transmission rate computed from the phenomenological contention model in (1) using previous slot subchannel occupancy. These observation variables are retained to represent the vehicle-side communication interface, but the present implementation does not compute a SINR-based rate or a power-dependent uplink rate. This previous-slot interpretation of $R_{v,r}(t)$ ensures that the state observation does not depend on the current-slot action $a_{v}(t)$, thereby avoiding circular dependency between state and action selection. The temporal sequence within each time slot t is therefore as follows: (i) the vehicle observes $s_{v}(t)$ using previous-slot channel/load indicators; (ii) the vehicle selects action $a_{v}(t)=[p_{v}(t),\;c_{v}(t)]$ according to its policy $\pi_{\theta_{v}}$; (iii) the environment transitions, producing the actual uplink rate, transmission delay, and queueing dynamics for slot t; (iv) the per-slot reward $r_{t}$ is computed at the end of the slot after the MEC layer has completed its decision (Section 4.3); (v) the trajectory is stored, and the next-slot observation $s_{v}(t+1)$ becomes available for the next iteration.

#### 4.1.2. Action Space of Vehicle

At each decision epoch, vehicle v outputs an interface action defined as

(18) $$a_{v}(t)=[p_{v}(t),\;c_{v}(t)],$$

where $p_{v}(t)$ denotes the transmission power of vehicle v at time slot t, and $c_{v}(t)$ represents the selected V2X uplink channel. Since the channel bandwidth $B_{c}$ is fixed, $c_{v}(t)$ determines the channel used for uplink transmission rather than the amount of allocated bandwidth. The power variable $p_{v}(t)$ is retained in the action representation for transmission-energy accounting through (11) and for compatibility with future power-dependent channel models. Under the present rate model in (1), however, $p_{v}(t)$ does not affect the realized uplink rate. The channel variable $c_{v}\left(t\right)$ determines the subchannel index and therefore the contention count $n_{c}(t)$, which affects the realized uplink rate and uplink delay through (1) and (2). Consequently, only the subchannel-assignment component affects the uplink rate in the present model, whereas the power component affects the transmission energy term in (11).

#### 4.1.3. Learning Strategy of Vehicle

Due to the simultaneous learning of multiple vehicle agents, the vehicle-side communication interface learning problem exhibits non-stationary dynamics when vehicles are trained independently. To mitigate this issue, MAPPO is adopted. MAPPO follows a centralized training with decentralized execution paradigm, where a centralized critic has access to the joint state information of all vehicles, including aggregate subchannel occupancy and RSU congestion states. This centralized critic enables more accurate value estimation by accounting for interactions among vehicle agents, thereby stabilizing policy learning and accelerating convergence. During execution, each vehicle independently applies its trained policy using only local observations, which avoids excessive signaling overhead and improves scalability as the number of vehicles increases. Accordingly, the detailed vehicle-side MAPPO-based communication interface procedure is summarized in Algorithm 1.

### 4.2. Edge-Side Learning for Model-Aware Computation

At the MEC layer, computation control is performed in a centralized manner because workload, queue, and resource state information can be aggregated by the SDN controller. In the proposed HMSC framework, a centralized SAC agent operates under SDN orchestration and performs system-level AI model selection and computational resource allocation based on state information collected from multiple RSUs and MEC servers.

#### 4.2.1. State Representation of MEC

The state observed by the centralized SAC agent at the MEC layer at time slot t is defined as

(19) $$s_{e}(t)=[Q_{e}(t),\;U_{e}^{CPU}(t),\;\lambda_{e}(t),\;T_{e}^{queue}(t)],$$

where $Q_{e}(t)$ denotes the task queue length at MEC server e, $U_{e}^{CPU}(t)$ represents the CPU utilization level of MEC server e, $\lambda_{e}(t)$ is the task arrival rate, and $T_{e}^{queue}(t)$ denotes the average queueing delay. These state variables are aggregated under SDN orchestration and provide the centralized agent with information on workload intensity, resource availability, and congestion conditions at the MEC layer.

The state representation $s_{e}(t)$ deliberately excludes per-task characteristics such as individual task data size, deadline, and per-model computational requirement. This design reflects the system-level operational scope of the centralized SAC agent under SDN orchestration: rather than producing a separate decision for each task instance, the agent determines an aggregate model-selection policy and resource-allocation policy for the task batch arriving in the current time slot, with per-task model assignment $m_{i}(t)$ and CPU allocation $f_{e,i}(t)$ derived from the aggregate policy via SDN dispatch. This separation between system-level policy learning and per-task dispatch enables the agent to scale with task arrival rate without inflating the input dimensionality, which is essential in vehicular environments where the number of concurrent tasks varies by an order of magnitude across density levels.

#### 4.2.2. Action Space of MEC

The centralized SAC agent at the MEC layer selects an action defined as

(20) $$a_{e}(t)=[m(t),\;f_{e}(t)],$$

where $m\left(t\right)\in \{L,\;H\}$ denotes the AI model class selected for time slot t, and $f_{e}(t)$ denotes the DVFS frequency level selected for slot t. The per-task assignments follow directly as $m_{i}(t)=m(t)$ and $f_{e,i}(t)=f_{e}(t)$ for all tasks i arriving in slot t, dispatched by the SDN controller; this constitutes the dispatch rule that maps the system-level decision to individual tasks without requiring per-task state observation. The action jointly determines the inference model type and the computational resource assigned to each task, thereby directly affecting the computation delay and energy consumption defined in Section 3.3.

Hybrid action parameterization. The MEC action $a_{e}(t)$ combines a discrete model selection $m\left(t\right)\in \{L,\;H\}$ and a continuous DVFS allocation via a hybrid head: (i) a Categorical head over the two model classes produces m(t); (ii) a tanh-squashed Gaussian head produces a value discretized to the nearest DVFS level $f_{e}\left(t\right)\in \left\{1,\;2,\;3,\;4,\;5\right\}$ GHz. The MEC controller issues a single (model, frequency) decision per slot, applied to all tasks arriving in that slot; per-task heterogeneity arises from queueing order and arrival timing rather than from per-task actions. Since concurrency is bounded by $N_{core}$ and all concurrent tasks share one frequency level, C2 holds by construction $\left(N_{core}\times f^{max}=F_{e}^{max}\right)$ without post hoc normalization.

#### 4.2.3. Learning Strategy of MEC

To handle the hybrid action space and the exploration demands of dynamic workloads, the MEC-side controller is implemented as a SAC agent with twin Q-critics and an entropy temperature $\alpha_{ent}$. In our implementation, the SAC update is performed online at each slot using the most recent transition (s, a, r, s′), so that the MEC policy remains coordinated with the vehicle-side MAPPO policies during joint training.

### 4.3. Training and Execution Procedure

**Figure 3:**
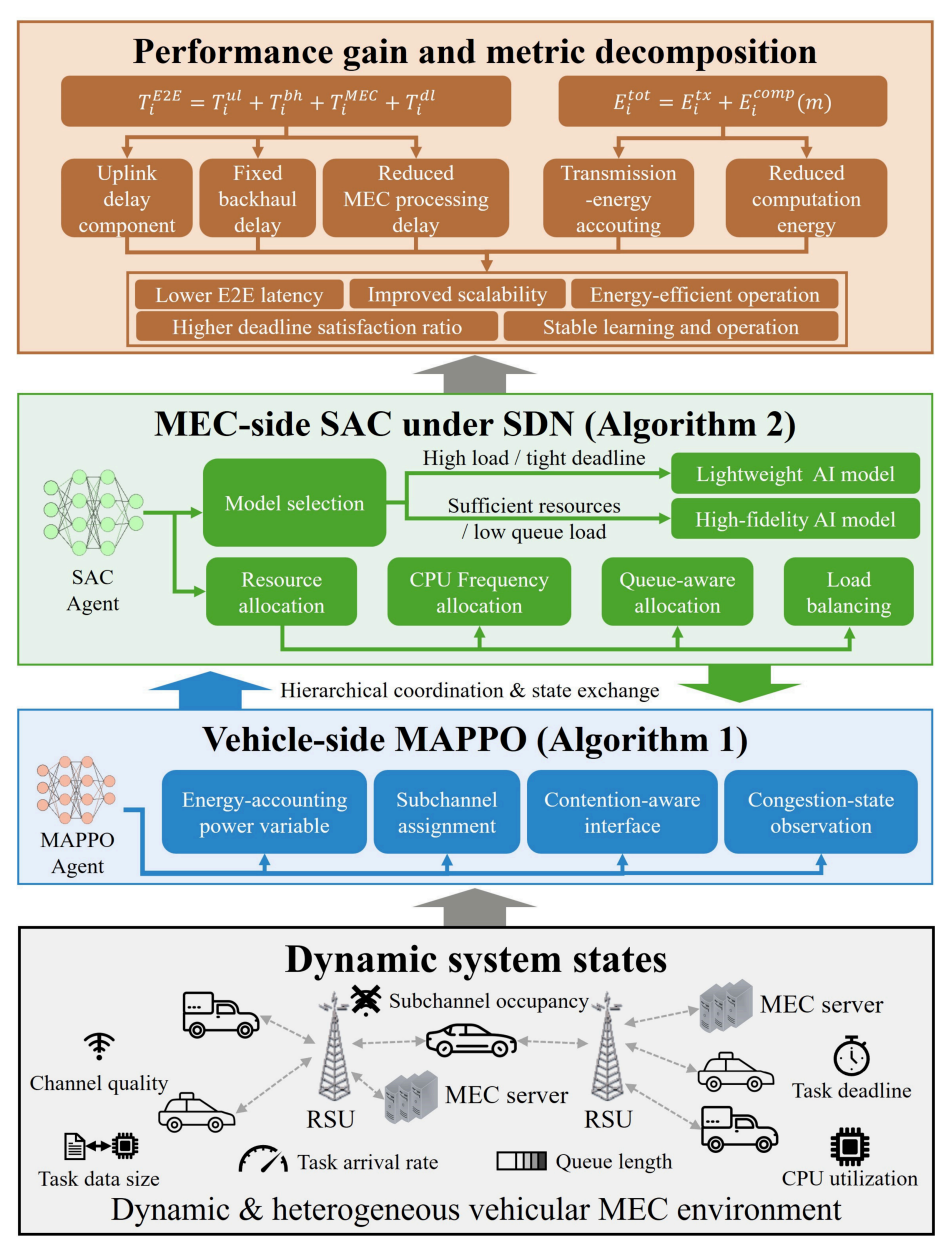
Training and execution flow of the proposed hierarchical DRL framework, emphasizing MEC-side model-aware computation control under a common vehicle-side communication interface.

Figure 3 illustrates the bottom-up training and execution flow of the proposed hierarchical DRL framework, where dynamic system states are progressively processed through vehicle-layer MAPPO and MEC-layer SAC agents to produce system performance gains. The figure highlights the sequential, rather than synchronous, interaction between the vehicle-side communication interface and the MEC-side computation controller under SDN orchestration within each time slot. Specifically, within each slot, the vehicle-side MAPPO policies are executed first to determine the interface actions and the resulting subchannel occupancy. The MEC-side SAC policy is then executed after the tasks arrive at the RSU/MEC side, using the aggregated MEC state to select the slot-level AI model and DVFS frequency. Thus, MAPPO and SAC are trained with a shared reward but executed in a sequential order within each time slot. During training, the MAPPO agents at the vehicle layer and the centralized SAC agent at the MEC layer interact with the simulated vehicular network environment in a coordinated manner. At each time slot, the vehicle agents first output vehicle-side interface actions based on their local observations. In the present implementation, these actions determine subchannel occupancy for the contention-based uplink model and provide the transmit-power variable used for energy accounting. Based on the aggregated workload, queue, and resource state information, the centralized SAC agent subsequently selects appropriate AI models and allocates computational resources for task processing. The resulting E2E delay, energy consumption, and deadline-violation indicator are jointly evaluated to compute a shared reward, defined as the negative composite cost in (16), which is used to update both the vehicle-layer MAPPO policy and the MEC-layer SAC policy. The shared per-slot reward $r_{t}=-J$ couples the two layers despite their differing update regimes. Experience trajectories are collected during policy rollouts and utilized for centralized training of the corresponding agents. As depicted in Figure 3, reward feedback is used only during the training phase to guide policy updates. No reward computation or parameter adaptation is performed during online execution. After training convergence, the learned MAPPO and SAC policies are fixed and deployed for online decision making under varying numbers of vehicles, stochastic task arrivals, and time-varying channel conditions.

Per-slot policy interaction sequence. To clarify the temporal coordination between the vehicle-layer MAPPO and MEC-layer SAC agents within each time slot t, we specify the sequence of operations as follows. **Step 1**: Each vehicle observes its local state $s_{v}(t)$ (using previous-slot channel statistics, as defined in Section 4.1.1). **Step 2**: Each vehicle outputs its interface action $a_{v}(t)$ via its MAPPO policy. **Step 3**: The selected subchannel indices determine the subchannel occupancy $n_{c}(t)$, and tasks generated by the vehicles traverse the V2X uplink, arrive at the RSU, and are forwarded to the MEC server. **Step 4**: The SDN controller observes the aggregate MEC state $s_{e}(t)$ and the centralized SAC agent selects $a_{e}(t)=[m(t),\;f_{e}(t)]$, which the SDN controller dispatches to all tasks arriving in slot t. **Step 5**: Tasks are processed at the MEC server, producing per-task outcomes $T_{i}^{E2E},\;E_{i}^{tot},\;1[T_{i}^{E2E}\leq T_{max}]$. **Step 6**: The shared reward $r_{t}=-J$ is computed from the slot’s completed task outcomes (Equation (16)). Tasks whose completion extends beyond their arrival slot are attributed to the reward of the slot in which they complete; expired tasks are recorded as violations in the slot in which expiration is detected. A small number of tasks that remain pending with positive deadline slack at the episode boundary are censored from completed-task latency statistics; to avoid bias from such completed-task-only metrics, we additionally report arrival-based completion, drop, throughput, and energy metrics in Table 3. Both layers’ transitions reference the identical task outcomes at the same slot index t. **Step 7**: The reward is broadcast to both layers. The vehicle-layer transition is stored in the on-policy trajectory buffer D, whereas the MEC-layer transition $\left(s_{e}(t),\;a_{e}(t),\;r_{t},\;s_{e}(t+1)\right)$ is consumed immediately by the per-slot online SAC update without a replay buffer, consistent with Algorithm 2; both transitions reference the identical completed-task outcomes at index t. At the end of each training episode, advantages are estimated using GAE and the MAPPO policy is updated via its clipped surrogate objective, while the SAC policy is updated per slot via its entropy-regularized objective. The shared reward construction ensures that both layers receive consistent learning signals reflecting the joint outcome of their coordinated decisions.

### 4.4. Discussion

The proposed hierarchical DRL framework provides several advantages in MEC-assisted vehicular networks. First, decomposing the framework into a vehicle-side communication interface and a MEC-side model-aware computation controller reduces policy-coupling complexity and improves learning scalability. Second, the MAPPO agents at the vehicle layer provide a decentralized interface for subchannel assignment and energy accounting, while the centralized SAC agent at the MEC layer ensures stable model-aware computation control under dynamic workload and resource variations. Finally, by distributing vehicle-side interface decisions across MAPPO agents and coordinating MEC-side computation decisions through the centralized SAC agent, the proposed framework can be extended to dense vehicular scenarios with a manageable increase in computational complexity. In the present rate model (Equations (1) and (2)), the uplink rate is independent of transmit power, and the symmetric snapshot setting admits near-balanced subchannel loading. Therefore, the vehicle-layer policy mainly provides a consistent communication- interface representation and subchannel-load balancing mechanism, while the dominant latency and reliability gains in this evaluation arise from the MEC-side model-aware computation controller. Extending the communication model to a power- and SINR-dependent formulation is left for future work.

## 5. Performance Evaluation

### 5.1. Simulation Environment and Implementation Details

We developed a discrete-time simulation framework to jointly emulate V2X communication, MEC-based AI inference, and hierarchical DRL-based control processes. The simulation focuses on a representative single-RSU MEC scenario, where one RSU is connected to one MEC server equipped with four CPU cores. The number of vehicles is varied as 100, 300, 500, and 1000 to emulate different traffic-density levels in a snapshot-based evaluation setting. The single-cell controlled-evaluation setup is intentionally adopted to isolate the effects of vehicle-side communication contention and MEC-side workload contention on the performance of the proposed hierarchical control framework, without the confounding effects of inter-MEC migration, service handover, and multi-cell scheduling. This methodological choice enables a clean characterization of the fundamental behavior of fidelity-aware hierarchical control across varying vehicle densities, channel conditions, and uplink-throughput regimes. Real-world multi-cell deployments involve additional control-plane complexities that are orthogonal to the central contribution of this paper; their integration is discussed in Section 5.6 (scalability considerations) and is left for future investigation. Vehicle mobility is approximated through snapshot density variations rather than explicit trajectory traces; each time slot represents a static density snapshot used to evaluate communication and computation contention under different load levels, since the focus of our evaluation is steady-state performance rather than transient handover effects. Task arrivals are generated according to a Bernoulli process at each time slot, which provides a discrete-time approximation of Poisson task arrivals. The task arrival rate is varied from 0.1 to 0.5 tasks/vehicle/s depending on the simulation scenario. Each generated task is characterized by an input data size, a result data size, a latency deadline, and a model-dependent CPU-cycle requirement. The MEC server performs AI inference using either a lightweight model or a high-fidelity model, corresponding to $30\times 10^{6}$ and $100\times 10^{6}$ CPU cycles per task, respectively. The communication model uses four OFDM sub-channels for V2X uplink access. The effective uplink throughput values reported in this paper (default 80 Mbps; sweep range 40–120 Mbps in Section 5.5.2) refer to the per-channel aggregate uplink capacity that is shared by vehicles assigned to that channel across the K=4 OFDM sub-channels. With K=4 OFDM sub-channels, the corresponding system-level total uplink capacity ranges from 160 Mbps to 480 Mbps. For all evaluated schemes, uplink transmission operates under a common configuration: the vehicle-side transmit power settles at a fixed operating point of approximately 0.141 W (21.5 dBm), and subchannel assignment yields balanced per-subchannel contention across the K=4 OFDM subchannels; consequently, the realized uplink rate and transmission energy are statistically identical across schemes, so the reported performance differences arise solely from the MEC-side decisions under study. Accordingly, the vehicle-side MAPPO component should not be interpreted as providing transmission-power optimization gains in the current experiments; instead, it serves as a vehicle-side interface within the hierarchical architecture, while the measured performance gains are produced by MEC-side model selection and DVFS control. The downlink throughput is fixed at 100 Mbps, and the backhaul connection between the RSU and MEC server is modeled as a constant 5 ms delay. Channel conditions are represented by an effective scaling factor η, where η=1.0 denotes the normal condition and η=0.5 denotes the degraded condition. The MAPPO and SAC policies are trained for 500,000 environment steps before evaluation. All reported results are averaged over five independent random seeds. Unless otherwise specified, the simulation parameters are set to the default values summarized in Table 2.

### 5.2. Baseline Schemes

To evaluate the effectiveness of the proposed HMSC framework, we compare its performance with three representative baseline schemes that reflect different design philosophies, ranging from deterministic rule-based heuristic policies to non-hierarchical learning. All baseline schemes are evaluated under identical settings on the number of vehicles, task generation processes, channel conditions, and performance metrics to ensure a fair comparison. The Fixed-Light scheme is a deterministic rule-based heuristic baseline in which all tasks are processed using the lightweight AI model throughout the simulation, regardless of network dynamics or computation load. This scheme represents a conservative low-complexity rule that prioritizes low computation cost and serves as a lower-bound reference for energy consumption. The Fixed-High scheme is another deterministic rule-based heuristic baseline that processes all tasks using the high-fidelity AI model at the MEC server. Although this rule can provide higher inference accuracy, it incurs higher computation latency and energy consumption, representing a static high-complexity heuristic that ignores dynamic queue, load, and resource conditions. These two fixed-model schemes are included as easily implementable non-learning heuristic references. They require no training, no neural network inference, and no online policy update, and therefore represent practical static rules that can be directly deployed when system designers prioritize implementation simplicity. However, because they cannot adapt their model-selection behavior to the current MEC queue state, CPU utilization, task arrival intensity, or deadline pressure, they provide limited robustness under time-varying workload conditions. The comparison with these deterministic heuristic baselines is therefore used to clarify the necessity of DRL-based MEC-side model-aware control. The Flat-DRL scheme employs a single centralized PPO agent that performs non-hierarchical control, without the vehicle-layer/MEC-layer decomposition adopted in the proposed framework. Its state is a fixed five-dimensional aggregate $s(t)=[t,\;{channel}_{mode},\;Q_{norm},\;Q_{len},\;slack]$ that is independent of the number of vehicles V; consequently, a single network is applied unchanged across all evaluated vehicle densities, and no vehicle-order fixing, padding, masking, or per-density retraining is required. Its action $a(t)=[m(t),\;f_{e}(t)]$ is likewise density-independent, selecting the AI model class m(t)∈{L, H} and the DVFS frequency level $f_{e}(t)$ for the current slot; vehicle-side transmission power and channel selection are not part of this baseline’s action space and are held at a fixed default configuration identical across all scenarios. The continuous DVFS component is parameterized by a tanh-squashed Gaussian head and the discrete model component by a Categorical head, using the same network architecture (two hidden layers of 256 units each) and training budget (learning rate $3\times 10^{-4},\;{GAE}_{\lambda }=0.95,\;{clip}_{\varepsilon }=0.2,\;K=5$ epochs, 500,000 environment steps) as the proposed framework. This baseline is not intended to represent a full joint communication computation baseline with the same vehicle-side action space as HMSC. Instead, it serves as a MEC-side flat-learning baseline designed to compare the proposed MEC-side SAC controller with a non-hierarchical PPO controller under the same common uplink configuration. Because all evaluated schemes share the fixed vehicle-transmission setting described in Section 5.1, the performance gap between Flat-DRL and HMSC should be interpreted as the benefit of MEC-side model-aware control rather than evidence of vehicle-side transmission control superiority. In contrast, the proposed HMSC framework combines a MAPPO-based vehicle-side communication interface with SAC-based adaptive AI model selection and computational resource allocation at the MEC layer under a unified composite objective that captures latency, energy, and deadline satisfaction.

### 5.3. Evaluation Metrics

To comprehensively evaluate the proposed framework, we adopt three performance metrics that jointly capture communication efficiency, computation cost, and service reliability. The average E2E latency measures the total delay experienced by a task from its generation at the vehicle to the return of the inference result, and it is computed by averaging the per-task E2E delay defined in (10), as formulated in (13). The average energy consumption quantifies the total energy cost incurred for task processing, including both uplink transmission energy and DVFS-based computation energy, and is obtained by averaging the per-task total energy defined in (12), as formulated in (14). The DSR represents the fraction of tasks completed within their latency deadlines, as defined in (15), and reflects the service reliability of the system under dynamic traffic and network conditions. Completed-task latency and completed-task energy are averaged over tasks that are completed within the simulation horizon, whereas DSR, completion rate, drop rate, throughput, and arrival-based energy are computed with respect to all arriving tasks. All reported values are averaged over five independent runs with different random seeds. It should be noted that the reported E2E latency values include all modeled delay components from task generation at the vehicle to result delivery, including uplink transmission, backhaul forwarding, MEC queueing, AI inference computation, and downlink response delays. Therefore, the reported latency values reflect the overall E2E delay, including communication, queueing, and computation overhead, rather than the delay of an individual subsystem component. Accordingly, the relative comparison among schemes is used as the primary indicator of framework efficiency.

### 5.4. Simulation Scenarios and Parameter Settings

To evaluate the robustness of the proposed framework under diverse network and workload conditions, we consider multiple simulation scenarios by varying the number of vehicles, task data size, task arrival rate, effective V2X uplink throughput, and channel condition. The number of vehicles is set to 100, 300, 500, and 1000 to control the offered traffic load and the resulting communication and computation contention in the single-RSU MEC scenario. The task data size is varied from 0.2 to 1.5 MB to reflect heterogeneous perception and decision-making tasks with different input data sizes. The task arrival rate is varied from 0.1 to 0.5 tasks/vehicle/s to evaluate the effect of workload intensity. The effective V2X uplink throughput is set to 80 Mbps by default and varied from 40 to 120 Mbps in the throughput sensitivity analysis. In addition, two representative channel conditions are considered: normal and degraded conditions. The degraded condition is modeled by setting the channel scaling factor to η=0.5, whereas the normal condition uses η=1.0. All scenario results are averaged over five independent runs with different random seeds.

### 5.5. Performance Evaluation Results

**Figure 4:**
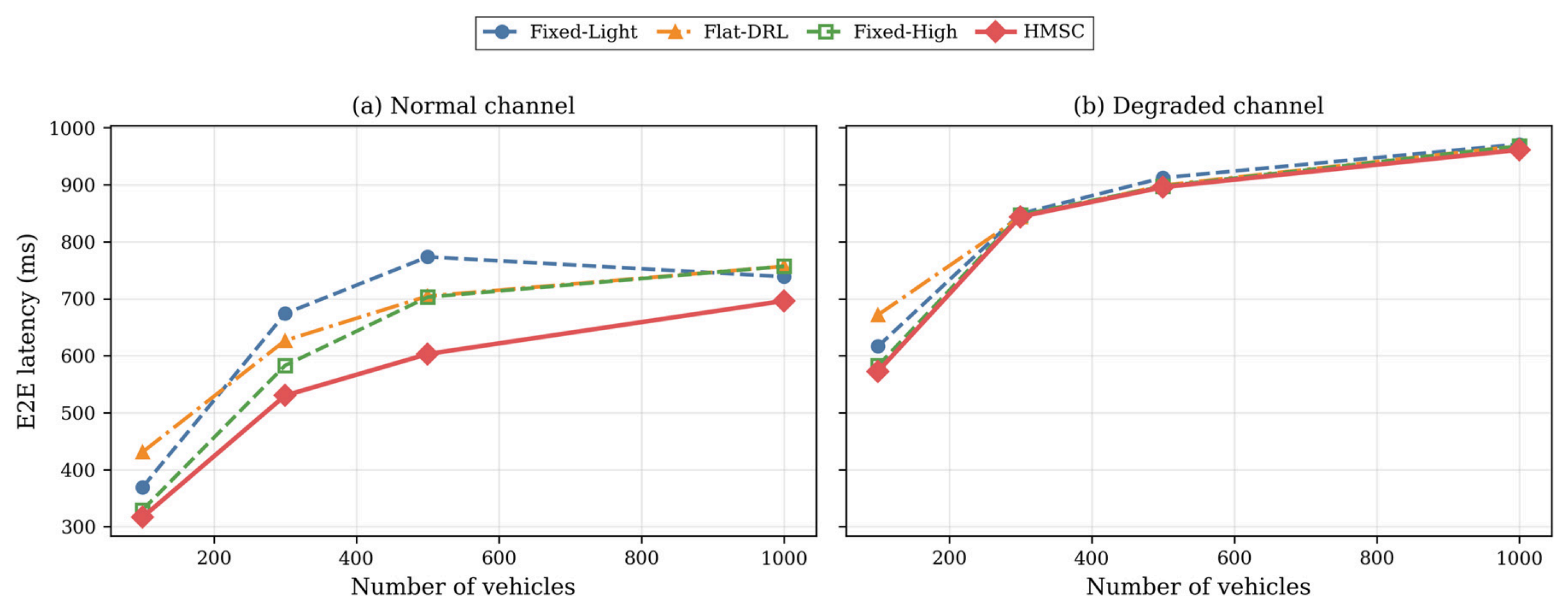
Average E2E latency under varying numbers of vehicles: (**a**) normal channel condition and (**b**) degraded channel condition.

**Figure 5:**
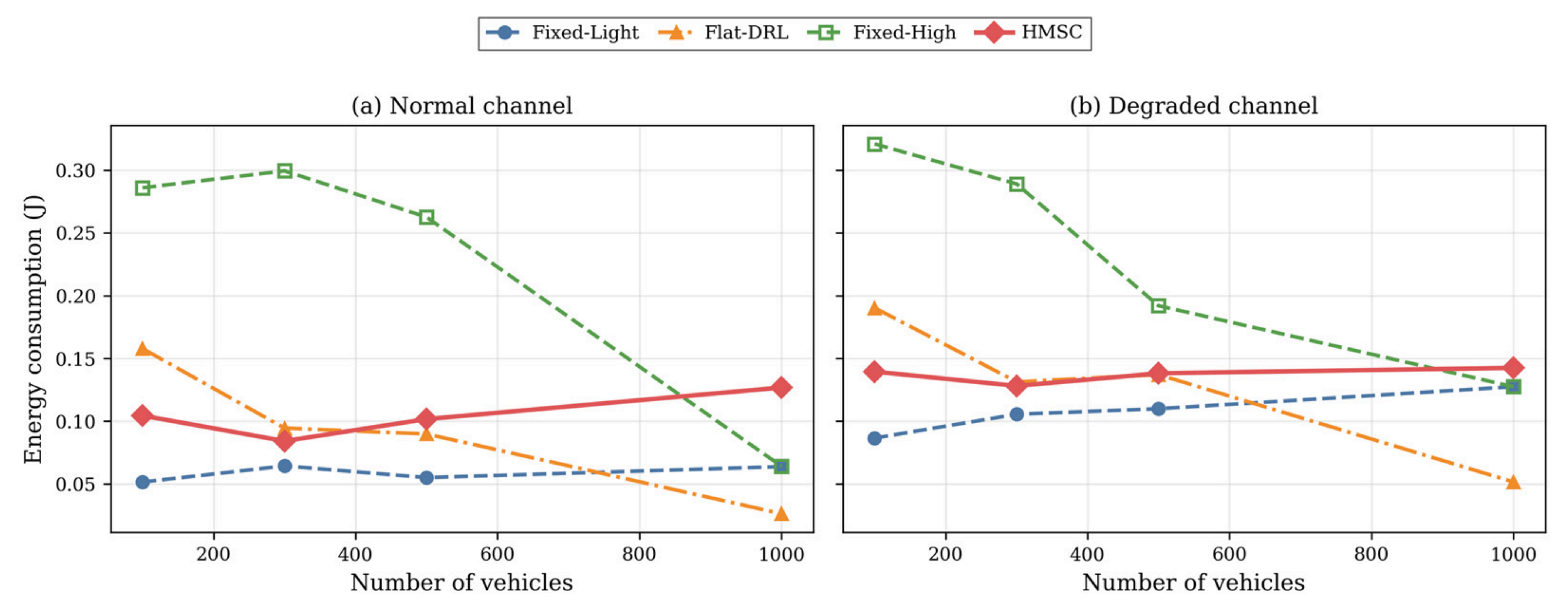
Average energy consumption under varying numbers of vehicles: (**a**) normal channel condition and (**b**) degraded channel condition.

**Figure 6:**
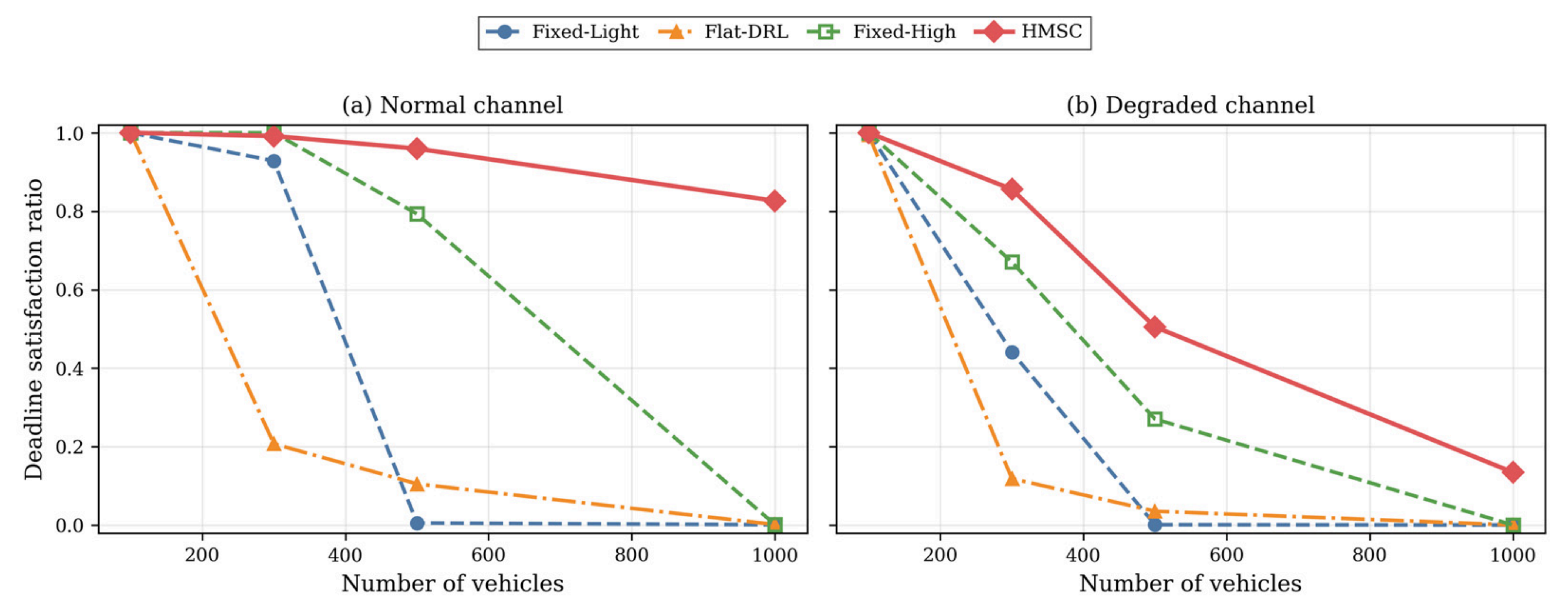
Deadline satisfaction ratio under varying numbers of vehicles: (**a**) normal channel condition and (**b**) degraded channel condition.

**Figure 7:**
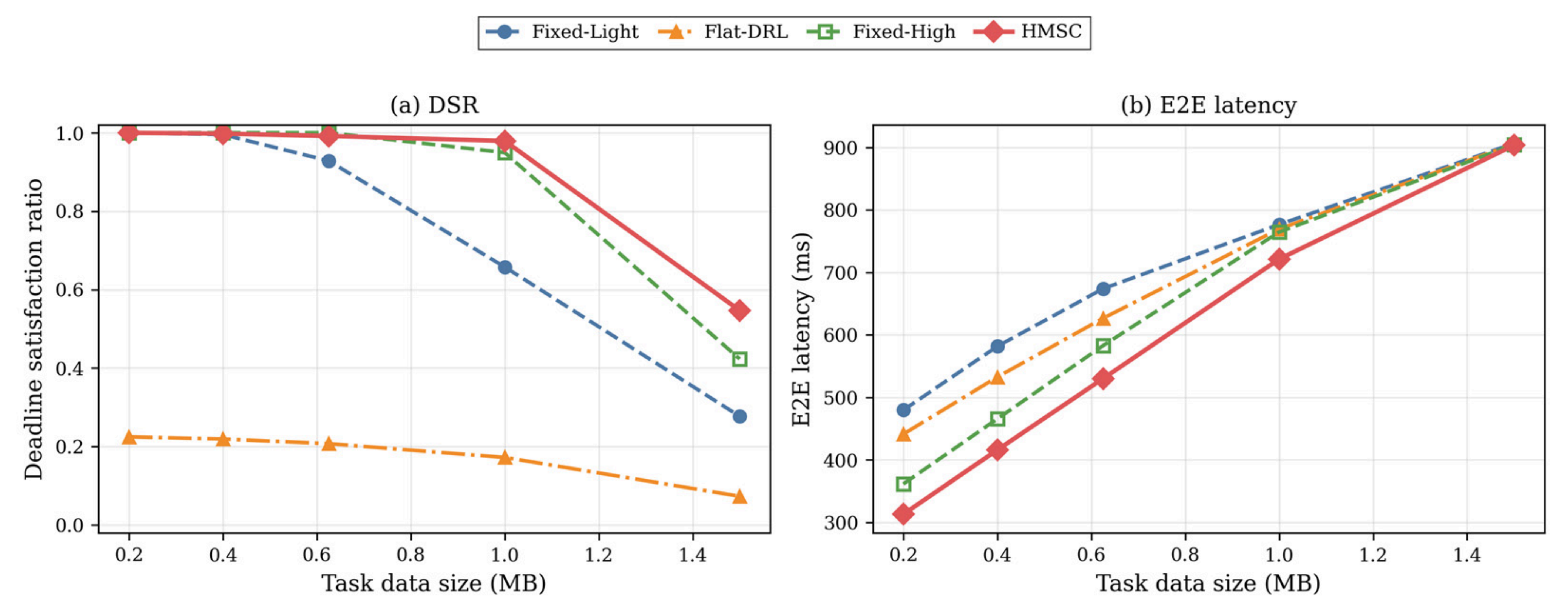
Impact of task data size on (**a**) DSR and (**b**) E2E latency under the normal channel condition.

**Figure 8:**
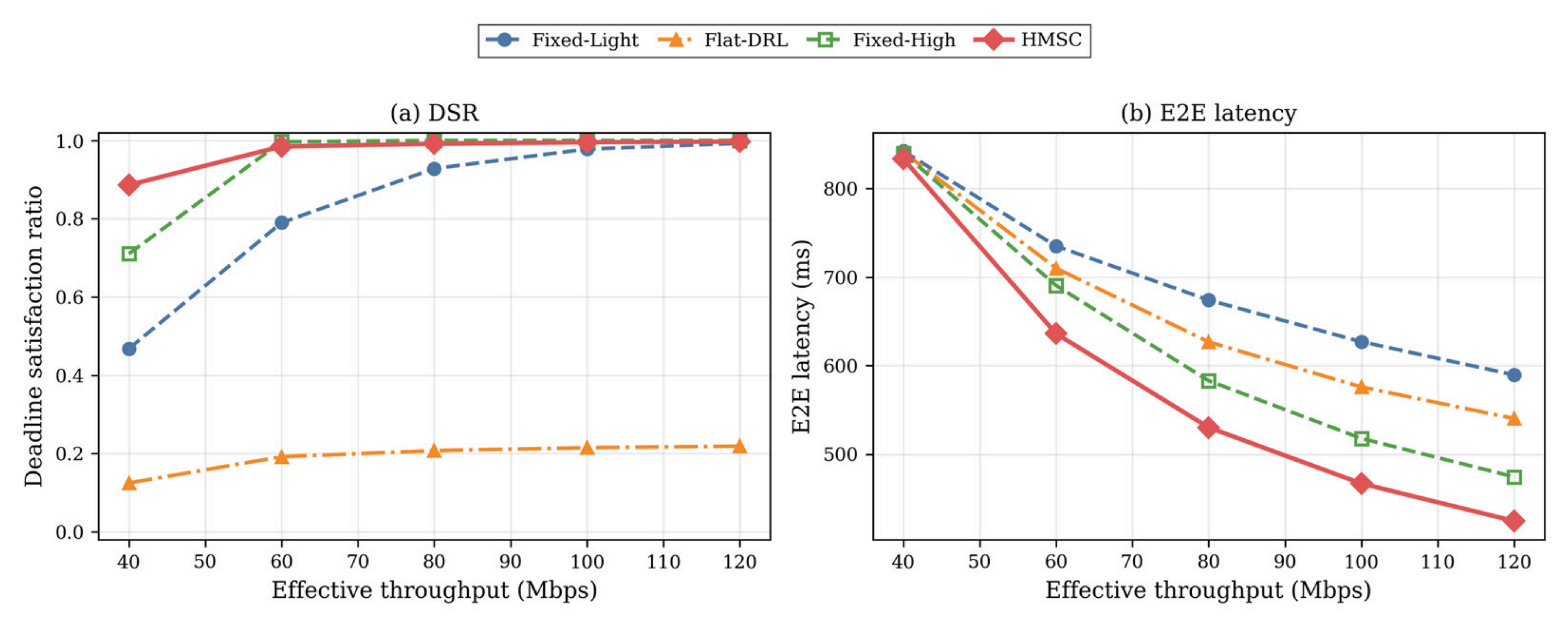
Impact of effective V2X uplink throughput on (**a**) DSR and (**b**) E2E latency under the normal channel condition.

**Figure 9:**
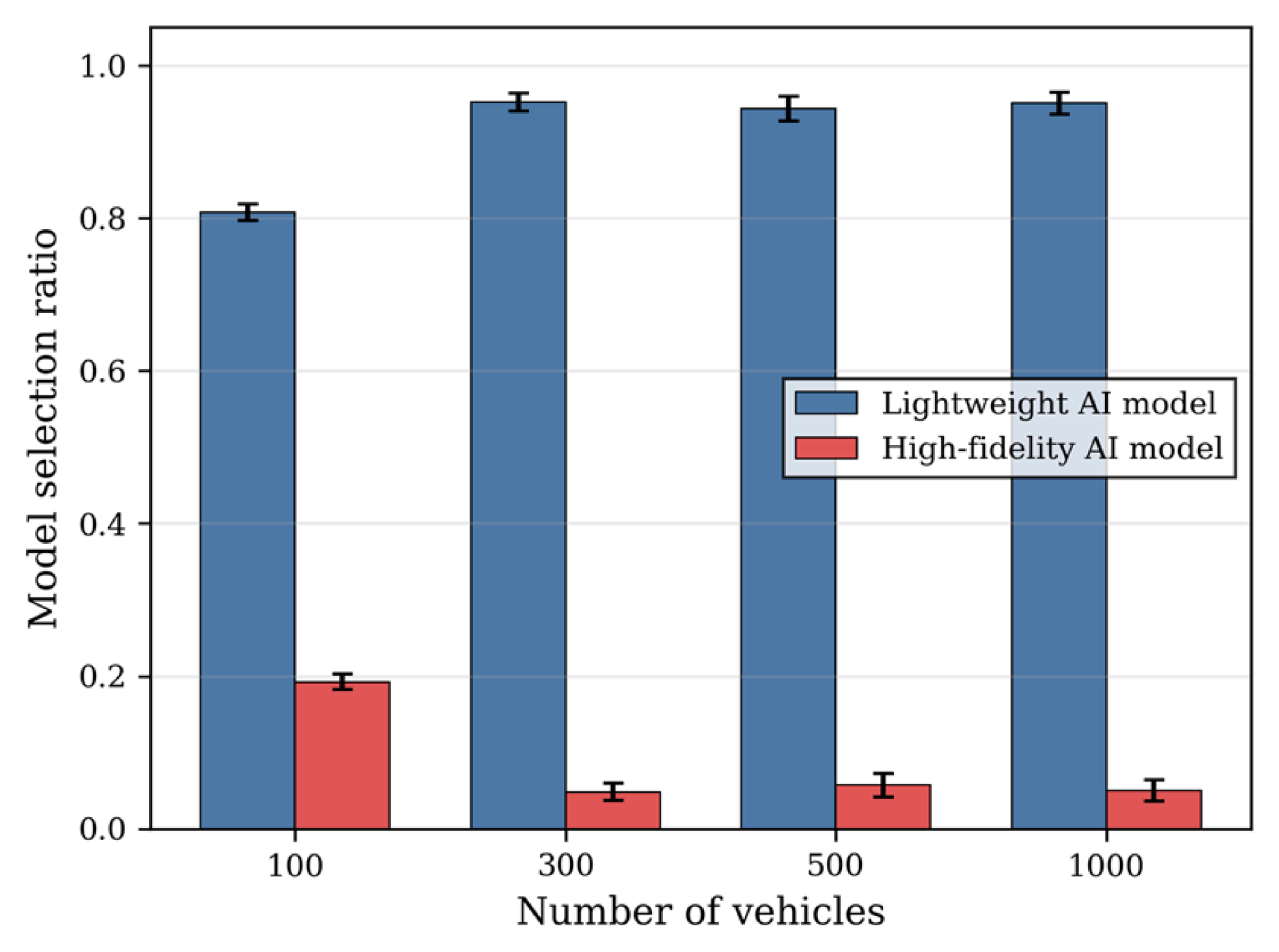
AI model selection ratio of the proposed HMSC framework under varying numbers of vehicles.

**Figure 10:**
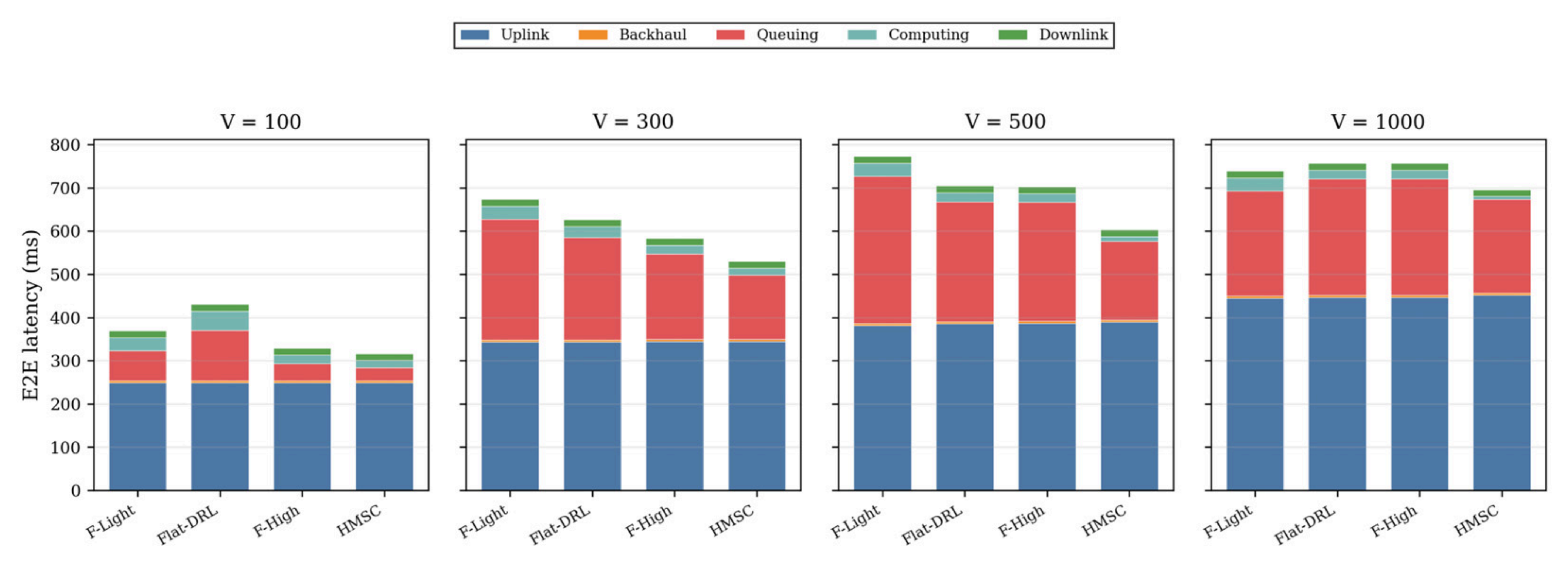
Breakdown of E2E latency into uplink transmission, backhaul forwarding, MEC queueing, computation, and downlink response delays for different numbers of vehicles.

**Figure 11:**
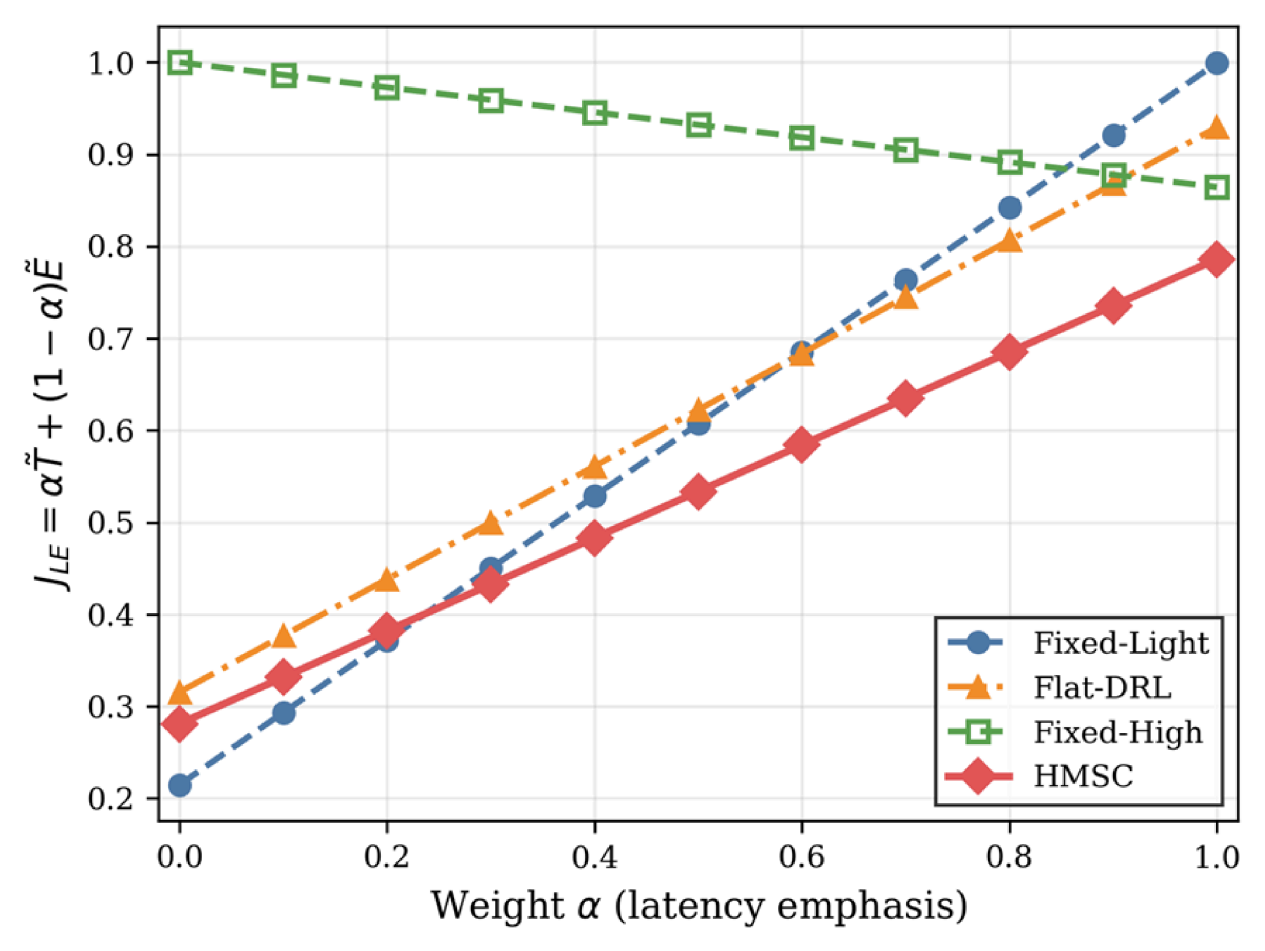
Normalized latency-energy sensitivity cost $J_{LE}$ under varying values of the weighting factor α.

In this subsection, we evaluate the proposed HMSC framework against three baseline schemes: Fixed-Light, Fixed-High, and Flat-DRL. The evaluation considers E2E latency, energy consumption, DSR, robustness under varying system parameters, and the underlying model-selection behavior. All reported results are averaged over five independent random seeds, and error bars in Figure 4, Figure 5, Figure 6, Figure 7, Figure 8, Figure 9, Figure 10 and Figure 11 denote one standard deviation across seeds. To quantify the statistical significance of the observed performance gaps, we performed paired two-sided *t*-tests between HMSC and each baseline on the primary metrics, including average E2E latency, DSR, and the weighted cost $J_{LE}$. Because multiple comparisons are conducted across baselines, metrics, and evaluation points, Holm–Bonferroni correction (α=0.05) is applied. After correction, the main performance gaps remain significant for most practically relevant congested regimes, especially for V≥300. At V=100, all schemes achieve near-complete task completion, so completion- and throughput-based comparisons provide limited discriminative information. In the most extreme degraded-channel high-density regime (V=1000 under η=0.5), the DSR values of all schemes are low, and the statistical discriminative power is correspondingly reduced. We also note that, since average latency and energy metrics are computed only over tasks that complete within the simulation horizon, the metric averaging is influenced by per-scheme task completion rate; we discuss this effect explicitly in the energy consumption analysis of Figure 5 (Section 5.5.1), where lower energy under baseline schemes at high density reflects the higher fraction of incomplete (queued or dropped) tasks rather than improved per-task efficiency.

#### 5.5.1. Overall Performance Under Varying Numbers of Vehicles

Figure 4 shows the average E2E latency as a function of the number of vehicles under normal and degraded channel conditions. As the number of vehicles increases, all schemes exhibit higher latency because of intensified V2X channel contention and increased queueing at the MEC server. Under the normal channel conditions, as shown in Figure 4a, the proposed HMSC framework consistently achieves the lowest E2E latency across the entire range of vehicle numbers. Its advantage becomes more pronounced in dense scenarios: at 1000 vehicles, HMSC achieves ≈696 ms over completed tasks, compared with 739 ms (Fixed-Light) and 759 ms (Fixed-High, Flat-DRL). This result indicates that MEC-side, model-aware control effectively mitigates computation-side congestion; because all schemes share common uplink conditions in this evaluation, the latency advantage of HMSC is attributable to this MEC-side coordination. Under the degraded channel condition, as shown in Figure 4b, the E2E latency increases more steeply for all schemes because the reduced effective throughput aggravates both uplink transmission delay and MEC queueing delay. Nevertheless, the proposed HMSC framework maintains a latency advantage at low-to-moderate numbers of vehicles, where adaptive model selection and MEC-side resource allocation can still effectively reduce congestion. At the highest number of vehicles, the performance gap becomes smaller as the system approaches a highly congested regime in which all schemes are constrained by limited communication capacity.

Figure 5 illustrates how the average energy consumption changes with the number of vehicles under normal and degraded channel conditions. As shown in Figure 5a, under the normal channel condition, the Fixed-Light scheme generally achieves the lowest energy consumption because it exclusively uses the lightweight AI model, whereas the Fixed-High scheme incurs higher energy consumption under low-to-moderate loads due to its continuous use of the high-fidelity model. The proposed HMSC framework maintains a balanced energy profile by adaptively selecting between lightweight and high-fidelity models according to the current communication and computation states. A consistent tendency is observed under the degraded channel condition, as shown in Figure 5b. At a high number of vehicles, the energy consumption of HMSC becomes higher than that of some baseline schemes. However, this result should be interpreted together with the DSR results in Figure 6. The lower energy consumption of the baseline schemes under highly congested conditions is mainly caused by their poor task completion performance: many tasks fail to satisfy their latency deadlines before completing useful inference, which reduces the accumulated computation energy. In contrast, HMSC successfully completes a larger fraction of tasks and therefore incurs the corresponding computation energy for productive inference. This indicates that the proposed framework does not merely minimize energy consumption but achieves a more reliable latency–energy trade-off by maintaining higher service reliability under dense traffic conditions. To ensure that the latency–energy comparison is not biased by differing completion rates, Table 3 reports arrival-based metrics.

The arrival-based view clarifies Figure 5: the apparently lower energy of baseline schemes at high density coincides with completion below 0.01 and drop above 0.99 at V=1000, reflecting dropped tasks rather than efficient inference. HMSC sustains the highest throughput (up to 248.1 tasks/s at V=1000) and completion, confirming its energy is spent on productive, deadline-satisfying inference. Compared with the deterministic rule-based heuristic baselines, HMSC provides a clear advantage because it adapts MEC-side model-selection and DVFS decisions to queue buildup, workload intensity, and deadline pressure, whereas the fixed heuristics cannot react to congestion or deadline violations.

Figure 6 presents the DSR as a function of the number of vehicles under normal and degraded channel conditions. As shown in Figure 6a, under the normal channel condition, the DSR of the baseline schemes decreases rapidly as the network load increases because more tasks violate their latency deadlines due to intensified channel contention and MEC queueing congestion. This degradation becomes more severe under the degraded channel condition, as shown in Figure 6b. Under this condition, the proposed HMSC framework sustains a higher DSR than all baseline schemes at low-to-moderate numbers of vehicles. At the highest load of V=1000, the DSR of all schemes decreases substantially because of the combined effects of channel degradation and severe congestion. Nevertheless, HMSC still achieves a DSR of 0.134, while all baseline schemes drop below 0.01. This result demonstrates that the proposed framework provides improved service reliability under dense and degraded network conditions. The robustness of HMSC stems from its MEC-side, model-aware control, which adaptively shifts toward lightweight inference and allocates computational resources to alleviate queueing congestion under heavy load.

#### 5.5.2. Impact of System Parameters on E2E Latency

Figure 7 investigates the impact of task data size on DSR and E2E latency under the normal channel condition. As shown in Figure 7a, the DSR of all schemes decreases as the task data size increases because larger input data sizes increase the uplink transmission delay and aggravate queueing pressure at the MEC server. Nevertheless, the proposed HMSC framework maintains the highest DSR across the entire range of task data sizes. Even at 1.5 MB, HMSC achieves a DSR of approximately 0.55, whereas the Flat-DRL baseline remains below 0.25 throughout the evaluated range. As shown in Figure 7b, the proposed HMSC framework achieves the lowest E2E latency at small-to-moderate task data sizes through MEC-side AI model selection and computational resource allocation. As the task data size approaches the upper bound, the latency of all schemes increases because the communication delay becomes dominant. Even in this regime, HMSC remains competitive with the baseline schemes, demonstrating its robustness under increasing input data sizes.

Figure 8 examines the effect of effective V2X uplink throughput on DSR and E2E latency under the normal channel condition. As shown in Figure 8a, increasing the uplink throughput improves the DSR of all schemes by alleviating uplink congestion. The proposed HMSC framework achieves the highest DSR under limited uplink-throughput conditions. For example, at 40 Mbps, HMSC achieves a DSR of 0.886, whereas Fixed-High and Fixed-Light achieve DSR values of 0.710 and 0.468, respectively. This result demonstrates the advantage of MEC-side adaptive model selection and computation control under constrained uplink conditions. At higher throughput levels, particularly above 80 Mbps, both HMSC and Fixed-High approach near-perfect DSR because the uplink bottleneck is largely alleviated. As shown in Figure 8b, the proposed HMSC framework achieves the lowest E2E latency across the entire throughput range, with the performance gap being most significant under limited uplink-throughput conditions. These results indicate that the proposed MEC-side model-aware controller remains effective under constrained uplink conditions while maintaining stable latency and service reliability.

#### 5.5.3. Mechanism and Learning Analysis

Figure 9 provides quantitative insight into the adaptive AI model selection behavior of the proposed HMSC framework under varying numbers of vehicles. At a low number of vehicles, V=100, the proposed policy selects the high-fidelity model for a non-negligible fraction of tasks, approximately 20%, indicating that the framework can exploit available computational headroom to use a more computation-intensive inference model when the system load is light. As the number of vehicles increases, the proportion of lightweight model selection rises to over 90%, showing that the framework progressively shifts toward computationally efficient inference to alleviate congestion under heavy load. This adaptive behavior demonstrates that the proposed framework learns a load-aware AI model selection strategy rather than relying on a static assignment, which helps explain the latency and service reliability improvements observed in the preceding results.

Figure 10 presents a breakdown of the E2E latency into its constituent components, including uplink transmission, backhaul forwarding, MEC queueing, computation, and downlink response delays, for different numbers of vehicles. For the baseline schemes, the queueing delay becomes the dominant contributor to the overall E2E latency as the number of vehicles increases, indicating severe congestion at the MEC server. In contrast, the proposed HMSC framework substantially reduces the queueing component through MEC-side AI model selection and computational resource allocation. Although some delay components, such as backhaul and downlink delays, are largely determined by fixed simulation settings, HMSC reduces the overall E2E latency by alleviating MEC queue buildup and selecting computationally efficient inference models under heavy load. This result indicates that the latency advantages of the proposed framework primarily originate from congestion-aware MEC-side coordination of model selection and resource allocation, rather than from optimizing a single delay component in isolation.

Figure 11 evaluates a normalized latency–energy sensitivity cost $J_{LE}$ under varying values of the weighting factor α, where $J_{LE}=\alpha \tilde{T}+(1-\alpha )\tilde{E}$. This metric is computed post hoc to examine the relative emphasis between E2E latency and energy consumption separately from the training objective J defined in (16). For α≥0.3, the proposed HMSC framework achieves the lowest $J_{LE}$ among all schemes, demonstrating its robustness across a wide range of latency–energy preferences. The Fixed-Light scheme attains a lower cost only when energy consumption is heavily emphasized, i.e., α<0.3, because it minimizes computation energy by exclusively using the lightweight AI model. However, this low-cost behavior should be interpreted together with its degraded latency and DSR performance, as shown in the preceding results. At the balanced operating point of α=0.5, which mirrors the equal latency–energy emphasis $w_{T}=w_{E}=1.0$ used in the training reward (excluding the deadline penalty term), the proposed HMSC framework achieves the most favorable normalized latency–energy cost by jointly reducing E2E latency and maintaining reliable task completion while avoiding excessive energy consumption. These results confirm that HMSC effectively adapts to different optimization preferences and provides a stable latency–energy trade-off across the evaluated weighting range. It should be noted that $J_{LE}$ in Figure 11 is computed using post hoc normalization across all compared schemes at each operating point, enabling a fair cross-scheme comparison of the latency–energy trade-off. This differs from the fixed-reference normalization ($T_{max}=1.0\;s,\;E_{ref}=0.3$ J) used in the training objective J defined in (16).

Finally, motivated by the control-plane latency discussion in Section 3.3, we quantify the sensitivity of HMSC to residual control-loop delay. The control loop includes vehicle-state collection (piggybacked on the uplink, within $T_{i}^{ul}$), RSU-to-controller backhaul (the 5 ms backhaul delay), controller processing (0.1–1 ms in typical SDN deployments [43]), and command delivery. To evaluate robustness to residual control latency $\delta_{ctrl}$, we add $\delta_{ctrl}\in \{0,10,50,100\}$ ms to recorded E2E latency, holding the trained policy fixed. Under normal conditions HMSC maintains DSR ≥ 0.985 for V≤300 even at 100 ms; degradation is sharp at V=1000 (mean completed-task latency ≈ 696 ms). Under degraded conditions the sensitivity is more pronounced: at V=500 the DSR falls from 0.530 (δ=0) to 0.261 (100 ms), and at V=1000 from 0.134 to near zero, as the enlarged uplink delay (η=0.5) already consumes most of the 1.0 s budget. The resulting DSR values under different residual control-loop delays are summarized in Table 4, indicating that $T_{max}$ should be co-designed with the expected control-loop latency.

#### 5.5.4. Inference Quality Implications of Adaptive Model Selection

While the proposed framework optimizes a composite latency–energy deadline objective and does not include inference accuracy as a decision variable, the load-aware model selection behavior shown in Figure 9 has direct implications for the resulting inference quality. To quantify these implications, we conduct a post hoc analysis using the representative accuracy values introduced in Section 3.3 ($A_{L}=0.457$ for the lightweight class and $A_{H}=0.673$ for the high-fidelity class, based on YOLOv5 family benchmarks). The expected average inference accuracy of the proposed framework is computed as

(21) $${\bar{A}}_{HMSC}=\rho_{L}(V)\cdot A_{L}+\rho_{H}(V)\cdot A_{H}$$

where $\rho_{L}(V)$ and $\rho_{H}(V)$ denote the lightweight and high-fidelity model selection ratios observed in Figure 9 at the vehicle density V. Table 5 reports the resulting expected accuracy under the four evaluated vehicle densities, alongside the corresponding accuracy attained by the static baseline schemes. Under the lightest load (V=100), HMSC retains approximately 19% high-fidelity selection, yielding an expected accuracy of 0.498, which is 4.1 percentage points higher than the Fixed-Light baseline (0.457). As congestion intensifies (V≥300), HMSC progressively shifts toward lightweight inference to preserve deadline satisfaction, converging to an expected accuracy of approximately 0.468.

This result reveals an important interpretation of the proposed framework’s behavior: HMSC implicitly trades a moderate fraction of inference accuracy (compared to a static high-fidelity policy) in exchange for substantial gains in deadline satisfaction ratio and energy efficiency, as documented in Figure 4, Figure 5 and Figure 6. The trade-off is asymmetric in the safety-critical vehicular context: a failed perception (deadline violation) provides no useful information to the downstream decision-making pipeline, whereas a slightly lower-fidelity result delivered within the deadline still supports timely vehicular control. The load-aware selection behavior of HMSC is consistent with this safety-critical priority. We note that a more refined approach in which inference accuracy itself becomes an explicit decision variable with continuous fidelity tuning, accuracy-aware reward shaping, and per-task accuracy constraints is a natural follow-up direction, as discussed in Section 6.

We emphasize the distinction between this analysis and accuracy-aware training. Equation (21) computes the accuracy induced by a policy trained without any explicit accuracy signal, whereas an accuracy-aware policy would receive model-specific accuracy in its reward or constraints and could learn different behavior. In this paper, accuracy is not incorporated into the training objective in order to isolate the effect of deadline-driven model selection on the latency–energy deadline trade-off. A meaningful accuracy-aware formulation requires a multi-level fidelity set with per-task accuracy constraints, which is developed as a follow-up direction in Section 6. We further note that the mAP values in Table 5 are measured on COCO val2017, whose object distribution and imaging conditions differ from onboard vehicular perception; therefore, they should be interpreted as relative fidelity indicators rather than deployment-grade accuracy values in driving scenes.

The results in Figure 4, Figure 5, Figure 6, Figure 7, Figure 8, Figure 9, Figure 10 and Figure 11 collectively demonstrate that the proposed HMSC framework consistently outperforms static inference policies and the non-hierarchical DRL approach in terms of E2E latency and service reliability, while achieving a balanced latency–energy trade-off. In addition, the proposed framework exhibits clear load-aware AI model selection behavior and stable performance across varying numbers of vehicles, task data sizes, effective V2X uplink throughput settings, and channel conditions. These results confirm the effectiveness of SDN-orchestrated, MEC-side model-aware control within the hierarchical framework for MEC-assisted vehicular networks.

### 5.6. Scalability Considerations

Although the experimental evaluation is conducted under a single-RSU MEC scenario, the proposed HMSC framework exhibits architectural properties that support extension to large-scale multi-cell deployments. We discuss three dimensions of scalability below.

#### 5.6.1. Vehicle-Layer Scalability

The MAPPO agents at the vehicle layer follow the centralized training, decentralized execution (CTDE) paradigm. During online execution, each vehicle independently applies its local policy based on the state $s_{v}(t)$, which depends only on local channel/load indicators and the serving-RSU congestion indicator. Therefore, the per-vehicle policy-inference cost, corresponding to the actor forward pass, is O(1) with respect to V. However, per-slot state collection and control messaging grow linearly with V, subchannel-occupancy aggregation scales with the co-channel vehicle count, and the centralized critic input during training grows with V. These were absorbed within the 1.0 s slot in our evaluation but constitute the practical scaling bottleneck; empirical multi-RSU/multi-MEC verification is left for future work.

#### 5.6.2. MEC-Layer Scalability

The centralized SAC agent at the MEC layer makes decisions based on aggregate state $s_{e}(t)=[Q_{e}(t),\;U_{e}^{CPU}(t),\;\lambda_{e}(t),\;T_{e}^{queue}(t)]$, which is computed by the SDN controller from per-MEC statistics rather than per-task observations. Consequently, the dimensionality of $s_{e}(t)$ is $O(\left|E\right|)$ where $\left|E\right|$ is the number of MEC servers, not $O(\left|I\right|)$ where $\left|I\right|$ is the number of tasks. This aggregation-based state representation enables the MEC-layer agent to scale gracefully with vehicle density, since the input dimensionality is bounded by the network topology rather than the workload intensity.

#### 5.6.3. Control-Layer Scalability

In multi-MEC deployments, the proposed SDN-based architecture can be hierarchically replicated: each local MEC controller manages an RSU cluster within a coverage region, while a higher-tier orchestrator coordinates inter-cluster service migration and load balancing. This two-tier organization is consistent with standard SDN deployment practices and limits the per-controller state space, mitigating signaling overhead. A detailed quantitative analysis of multi-MEC handover latency, service migration cost, and inter-controller signaling under the proposed framework is left for future investigation and is beyond the scope of the present paper, which focuses on establishing the fundamental performance characteristics of fidelity-aware hierarchical control.

## 6. Conclusions and Future Work

This paper proposed the HMSC framework for MEC-assisted vehicular networks, jointly optimizing E2E latency, energy consumption, and deadline satisfaction under heterogeneous channel conditions, varying numbers of vehicles, and time-varying task arrivals. Unlike conventional MEC optimization approaches that rely on fixed inference pipelines, the proposed framework explicitly integrates AI model selection into the MEC-side computation control loop, enabling adaptive switching between lightweight and high-fidelity AI models based on workload, queue, and resource states. To address scalability and non-stationarity challenges in dense vehicular environments, we design a hierarchical control architecture that separates the vehicle-side communication interface representation from MEC-side AI model selection and computation resource allocation. MAPPO agents at the vehicle layer provide a communication interface representation for subchannel assignment and energy accounting, while a centralized SAC agent at the MEC layer under SDN orchestration performs adaptive AI model selection and computational resource allocation. In the present evaluation, the primary performance gain is attributed to the MEC-side model-aware controller under the common uplink configuration, while richer power- and SINR-dependent communication control is left for future work. Thus, the proposed framework should be interpreted as a model-aware MEC orchestration framework evaluated under a simplified but explicit communication interface abstraction, rather than as a complete physical-layer communication control solution. The proposed framework was evaluated using a discrete-time simulation platform designed to capture V2X communication, task arrivals, MEC-based AI inference, and hierarchical DRL-based control under varying numbers of vehicles and workload conditions. The simulation results show that the proposed approach maintains robust performance across different numbers of vehicles, task data sizes, effective V2X uplink throughput settings, and channel conditions, while improving learning stability and scalability in highly loaded vehicular networks. These results indicate that coordinated MEC-side control of AI model selection and computation is critical to maintaining reliable and efficient vehicular services supported by MEC.

Building on the post hoc inference-quality analysis in Section 5.5.4, a natural follow-up direction is to extend the proposed framework along three axes that directly address the inference-quality dimension within the optimization formulation. First, the binary model selection m∈{L, H} can be generalized to a multi-level fidelity set $M=\{m_{1},\;m_{2},\ldots ,m_{K}\}$ spanning a representative model family, such as YOLOv5n/s/m/l/x. This extension can be implemented without changing the overall hierarchical architecture: the MEC-side SAC agent only needs to expand its categorical model-selection head from two classes to the K fidelity levels, while the DVFS head and the vehicle-side communication interface component can remain unchanged. Each model level $m_{k}$ would be associated with a profiled computation cost $C_{i}(m_{k})$, expected accuracy $A(m_{k})$, and energy model, enabling the SDN controller to dispatch a selected fidelity level and CPU frequency to the tasks arriving in each slot. For multi-stage inference pipelines, the same action representation can be further extended so that $m_{k}$ denotes either a single model fidelity level or a stage-depth configuration in a coarse-to-fine inference chain. Second, the cycle-based compute model can be replaced by a TFLOPS-aligned formulation that more faithfully reflects modern AI accelerators such as the NVIDIA Jetson AGX Orin. Third, accuracy-aware reward shaping and per-task accuracy constraints can be incorporated such that inference fidelity becomes a first-class MEC-side controllable resource alongside model selection and compute allocation. A separate future extension will consider a power- and SINR-dependent communication model in which transmission-power control can be evaluated explicitly. Beyond inference fidelity extensions, future research will also investigate cooperative perception across multiple vehicles, federated learning across distributed MEC domains, multi-MEC service migration and handover, and the integration of the proposed approach with emerging O-RAN and digital twin architectures, aiming to enable scalable and autonomous network control in next-generation vehicular systems.

## Figures and Tables

**Table 1:** Summary of Notations.

Notation	Description
v, i, r	Index of vehicle, task, RSU
$n_{c}(t)$	Vehicles on subchannel c
η	Channel-quality factor
$B_{UL}$	Per-subchannel uplink capacity
m∈{L,H}	AI model type (Lightweight or High-fidelity)
$D_{i}$	Input data size of task i
$C_{i}^{\left(m\right)}$	Required CPU cycles under model m
$R_{v,r}\left(t\right)$	Uplink transmission rate
$p_{v}\left(t\right)$	Transmission power of vehicle v at time t
$T_{i}^{ul}$	Uplink delay
$\tau_{bh}$	Fixed backhaul delay
$T_{i}^{bh}$	Backhaul delay
$T_{i}^{queue}$	Queueing delay at MEC
$f_{e,i}$	Allocated MEC CPU frequency
κ	DVFS energy coefficient
$T_{i}^{comp}\left(m\right)$	Computation delay
$T_{i}^{E2E}$	E2E delay
$E_{i}^{tx}$	Transmission energy
$E_{i}^{comp}\left(m\right)$	Computation energy
$E_{i}^{tot}$	Total energy
${\bar{T}}_{comp}$	Completed task average E2E delay
${\bar{E}}_{comp}$	Completed task average energy consumption
DSR	Deadline satisfaction ratio
$1\left(\cdot \right)$	Indicator function
$w_{T},\;w_{E}$	Latency and energy weights in the composite objective
β	Deadline violation penalty coefficient
$E_{ref}$	Reference energy bound
J	Composite training objective
$J_{LE}$	Post hoc latency–energy sensitivity cost
α	Weighting factor for latency–energy sensitivity analysis
$N_{core}$	Number of concurrent CPU cores at the MEC server
$F_{e}^{max}$	Maximum aggregate CPU frequency of the MEC server at RSU
$\delta_{ctrl}$	Residual control loop delay
ϵ	Maximum acceptable deadline-violation probability

**Table 2:** Simulation parameters.

Parameter	Value
Number of RSUs	1
Number of MEC servers	1
Number of MEC cores	4
Maximum aggregate MEC CPU capacity	20 GHz
Number of vehicles V	100, 300, 500, 1000
Vehicle model	Static density per time slot
Task arrival process	Bernoulli process per time slot
Task arrival rate λ	0.1–0.5 tasks/vehicle/s
Default task arrival rate λ	0.3 tasks/vehicle/s
Number of OFDM sub-channels K	4
Default effective V2X uplink throughput	80 Mbps
Effective V2X uplink throughput sweep	40, 60, 80, 100, 120 Mbps
Downlink throughput	100 Mbps
Backhaul delay	5 ms
Vehicle transmit-power action range $p_{v}(t)$	5–23 dBm (0.003–0.2 W)
Common transmit-power operating point	≈ 21.5 dBm (0.141 W)
DVFS levels $f_{e}$	1.0, 2.0, 3.0, 4.0, 5.0 GHz
Lightweight AI model $C_{L}$	$30\;\times \;10^{6}$ CPU cycles/task
High-fidelity AI model $C_{H}$	$100\;\times \;10^{6}$ CPU cycles/task
DVFS energy coefficient κ	0.5
Default task data size $D_{i}$	0.625 MB
Task data size sweep	0.2, 0.4, 0.625, 1.0, 1.5 MB
Result data size	0.1 MB
Task deadline $T_{max}$	1.0 s
Time slot duration Δt	1.0 s
Simulation duration	300 s (300 time slots)
Channel conditions	Normal: η=1.0; degraded: η=0.5
Latency weight $w_{T}$	1.0
Energy weight $w_{E}$	1.0
Deadline-violation penalty β	8.0
Energy normalization $E_{ref}$	0.3 J
Sensitivity weight α	0.0–1.0 (sweep)
[MEC agent-SAC]	
Algorithm	SAC (twin-Q, online update)
Entropy temperature $\alpha_{ent}$	0.2
Soft-update rate τ	0.005
Update scheme	Per-slot, batch size 1
Continuous head	Tanh-squashed Gaussian (DVFS-snapped)
Discrete head	Categorical (L/H)
Actor hidden dim	256
[Vehicle agent-MAPPO]	
Algorithm	MAPPO (PPO + centralized critic)
GAE parameter λ	0.95
Clipping parameter ε	0.2
Update epochs K	5
Continuous head	Tanh-squashed Gaussian
Discrete head	Categorical
Actor/critic hidden dim	128/256
[Flat-DRL-PPO]	
Algorithm	PPO (on-policy, clipped surrogate)
GAE λ/clip ε/epochs K	0.95/0.2/5
Hidden dim	256
[Shared]	
Learning rate	$3\times 10^{-4}$
Discount factor	0.99
DVFS levels	1.0, 2.0, 3.0, 4.0, 5.0 GHz
Total training steps	500,000
Random seeds	0, 1, 2, 3, 4

**Table 3:** Arrival-based metrics (normal channel, 5 seeds).

Scheme	V	E (J/Arrived)	Completion	Drop	Throughput (Task/s)
Fixed-Light	100/300/ 500/1000	0.052/0.064/ 0.055/0.064	1.000/0.928/ 0.005/0.001	0.000/0.072/ 0.995/0.999	30.0/83.4/ 0.8/0.2
Fixed-High	100/300/ 500/1000	0.286/0.300/ 0.263/0.064	1.000/1.000/ 0.793/0.001	0.000/0.000/ 0.207/0.999	30.0/89.9/ 118.8/0.4
Flat-DRL	100/300/ 500/1000	0.158/0.095/ 0.090/0.026	1.000/0.207/ 0.105/0.001	0.000/0.793/ 0.895/0.999	30.0/18.6/ 15.7/0.4
HMSC	100/300/ 500/1000	0.104/0.084/ 0.102/0.127	1.000/0.991/ 0.959/0.826	0.000/0.008/ 0.041/0.174	30.0/89.1/ 143.7/248.1

**Table 4:** DSR under control-loop delay $\delta_{ctrl}$ (HMSC, mean over 5 seeds).

Condition	V	0 ms	10 ms	50 ms	100 ms
Normal	100	1.000	1.000	1.000	1.000
	300	0.992	0.991	0.989	0.985
	500	0.959	0.958	0.952	0.944
	1000	0.826	0.825	0.819	0.780
Degraded	100	1.000	1.000	1.000	1.000
	300	0.857	0.835	0.729	0.589
	500	0.530	0.480	0.383	0.261
	1000	0.134	0.117	0.044	0.001

**Table 5:** Expected inference accuracy under varying vehicle densities (normal channel condition).

V	$\rho_{L}$	$\rho_{H}$	${\bar{A}}_{HMSC}$	${\bar{A}}_{Fixed-Light}$	${\bar{A}}_{Fixed-High}$
100	0.81	0.19	0.498	0.457	0.673
300	0.95	0.05	0.468	0.457	0.673
500	0.94	0.06	0.470	0.457	0.673
1000	0.95	0.05	0.468	0.457	0.673

## Data Availability

Derived data supporting the findings of this study are available from the corresponding author on request.
